# Single-cell RNA sequencing identifies CD8Teff cell activation as a predictive biomarker in triple-negative breast cancer immunotherapy

**DOI:** 10.1186/s43556-025-00306-2

**Published:** 2025-09-19

**Authors:** Luhui Mao, Zebang Zhang, Yongjian Chen, Qing Peng, Zhenjun Huang, Wenhao Ouyang, Dongqiang Zeng, Wei Ren, Zifan He, Tang Li, Zehua Wang, Ruichong Lin, Jianli Zhao, Jiannan Wu, Herui Yao, Yunfang Yu

**Affiliations:** 1 Guangdong Provincial Key Laboratory of Malignant Tumor Epigenetics and Gene Regulation, Guangdong-Hong Kong Joint Laboratory for RNA Medicine, Department of Medical Oncology, Sun Yat-Sen Memorial Hospital, Sun Yat-Sen University, Guangzhou, China; 2 Dermatology and Venereology Division, Department of Medicine Solna, Center for Molecular Medicine, Karolinska Institute, Stockholm, Sweden; 3 Department of Oncology, Nanfang Hospital, Southern Medical University, Guangzhou, Guangdong People’s Republic of China; 4 Faculty of Innovation Engineering, Institute for AI in Medicine and Faculty of Medicine, Macau University of Science and Technology, Taipa, Macao, China; 5 School of Computer and Information Engineering, Guangzhou Huali College, Guangzhou, China; 6 UMedEVO and UMedREVO Artificial Intelligence Technology (Guangzhou) Co., Ltd, Guangzhou, China; 7 Guangdong Provincial Key Laboratory of Cancer Pathogenesis and Precision Diagnosis and Treatment, AI Big Data Laboratory, Shenshan Medical Center, Memorial Hospital of Sun Yat-Sen University, Shanwei, China; 8 Department of Breast Surgery, The First Affiliated Hospital, Jinan University, Guangzhou, China

**Keywords:** Triple-negative breast cancer, CD8Teff cells, Single-cell RNA sequencing, C-X-C Motif Chemokine Ligand 13, Immunotherapy response, Artificial intelligence

## Abstract

Immunotherapy has emerged as a promising treatment option for triple-negative breast cancer (TNBC); however, the pronounced heterogeneity of the tumor immune microenvironment significantly hinders the prediction of therapeutic efficacy, with effective biomarkers also remaining limited. This study utilizes single-cell RNA sequencing (scRNA-seq) and transcriptome analysis to dissect the immune landscapes of TNBC, emphasizing CD8 effector T cells (CD8Teff) as potential predictors of immune checkpoint inhibitors (ICIs) treatment response. Immune profiling revealed that CD8Teff cells were predominantly enriched in "hot" tumors strongly correlating with improved progression-free and overall survival. Notably, the cytokine C-X-C Motif Chemokine Ligand 13 (CXCL13) emerged as a key regulator, with its high expression marking an immune-active tumor microenvironment favorable to ICI efficacy. Comprehensive analysis uncovered critical interactions between CD8Teff cells and other immune and stromal components, supporting the formation of an immunologically active tumor microenvironment (TME) conducive to tumor control. Additionally, metabolic reprogramming of CD8Teff cells in responsive tumors highlighted pathways that may influence ICIs efficacy. CD8Teff cells significantly influence the TME and predict ICI success in TNBC, with CXCL13 as a pivotal modulator. A pathology-based artificial intelligence model for CD8Teff recognition was developed, achieving an area under the curve (AUC) of 0.823 in the training cohort and 0.805 in the validation cohort. This study provides an in-depth characterization of the TNBC immune microenvironment, identifying CD8Teff functionality, CXCL13 signaling, and immune-metabolic pathways as critical determinants of immunotherapy success. These findings support the development of personalized treatment strategies based on immune profiling to enhance therapeutic efficacy in TNBC.

**Supplementary Information:**

The online version contains supplementary material available at 10.1186/s43556-025-00306-2.

## Introduction

Triple-negative breast cancer (TNBC) is a clinically aggressive subtype charscterized by poor prognosis and limited treatment options compared to other breast cancer subtypes [1, 2], necessitating the exploration of novel therapeutic approaches. Recent advances in immunotherapy, particularly immune checkpoint inhibitors (ICIs) targeting the PD-1/PD-L1 axis, have opened new avenues for treating TNBC by enhancing anti-tumor immune responses [3, 4]. The KEYNOTE-522 study demonstrated that adding pembrolizumab to neoadjuvant chemotherapy significantly improved event-free survival in early-stage TNBC, ICIs in combination with chemotherapy have been shown to increase the pathological complete response (pCR) rate by 4–17%, and improve 3-year event-free survival rates by 8–13%. Which establishing a foundational framework for integrating immunotherapy into standard treatment regimens [5]. Furthermore, immunotherapy has demonstrated considerable efficacy in advanced-stage TNBC, IMpassion031 and PCD4989g showed significant improvements in progression-free survival (PFS) and overall survival (OS), particularly among patients exhibiting high levels of tumor-infiltrating immune cells and increased immunogenicity [6–8]. However, response rates to ICIs remain highly variable, underscoring the urgent need for reliable biomarkers to predict which patients are most likely to benefit from these therapies.

The effectiveness of immunotherapy in breast cancer is closely tied to the characteristics of the tumor microenvironment (TME) [9]. Tumors are often categorized as "hot" or "cold" based on immune cell infiltration and activity, with hot tumors showing greater responsiveness to immunotherapy [10–12]. Currently, the classification of "hot" and "cold" tumors is primarily based on the assessment of CD8 + T cells and CD4 + T cells via immunohistochemistry (IHC). The IMpassion130 study assessed the efficacy of atezolizumab in combination with nab-paclitaxel for advanced TNBC patients, with its exploratory analysis revealing that CD8 + T cell infiltration in the tumor microenvironment could serve as a predictive biomarker for OS benefits from atezolizumab treatment in metastatic TNBC cases. Similarly, the PCD4989g trial assessed atezolizumab efficacy in various solid tumors, finding that patients with high CD8 + T cells expression had longer progression-free survival (irPFS) and OS [6–8]. However, the immune landscape within TNBC is highly heterogeneous, and the mere presence of CD8 + T cells does not consistently correlate with favorable therapeutic outcomes. This suggests that additional factors, including the functional status of CD8 + T cells and their interactions with other immune and stromal components within the TME, are critical determinants of immunotherapy efficacy [13, 14].

The advent of single-cell RNA sequencing (scRNA-seq) has enabled high-resolution profiling of the immune landscape within tumors, uncovering substantial heterogeneity among immune cell subsets. This technology has revealed that CD8Teff cells exhibit diverse functional states and dynamic interactions with other immune and stromal cells, which may dictate tumor immunogenicity and response to ICIs [15]. However, the complex network of immune crosstalk and its role in modulating immune checkpoint response remains incompletely understood. Furthermore, current definitions of "hot" and “cold” tumors, often based on static immune cell staining, fail to capture the dynamic and functional intricacies of the TME, limiting the precision of immune-based classification and treatment decisions.

This study leverages scRNA-seq and integrative multi-omics analyses to dissect the immune landscape of TNBC patients undergoing ICIs therapy, with a particular focus on CD8Teff dynamics. The functional and spatial characteristics of CD8Teff cells within the TME are key determinants of responsiveness to ICIs. By correlating single-cell immune profiles with clinical outcomes, we identified CD8Teff cells as central orchestrators of anti-tumor immunity, strongly associated with "hot" tumor phenotypes and favorable therapeutic responses. Furthermore, CD8Teff cells were found to modulate the TME through cytokine signaling via CXCL13 (C-X-C Motif Chemokine Ligand 13), metabolic reprogramming, and interactions with dendritic cells and stromal elements. To translate these findings into clinical practice, we developed an AI-based pathological model to classify "hot" and "cold" tumors based on CD8Teff distribution, achieving strong predictive performance. Collectively, our findings establish CD8Teff functionality as a critical biomarker for immunotherapy stratification and lay the groundwork for personalized immune-based strategies in TNBC.

## Result

### Immune cell profiles and dynamics in response to ICI treatment in TNBC

**Fig. 1:**
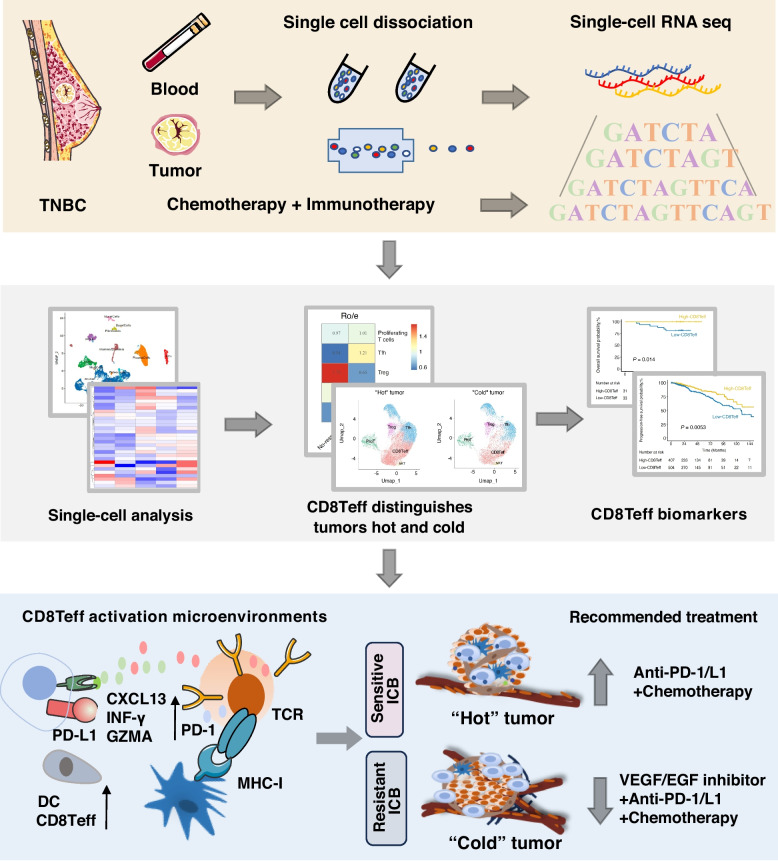
Workflow of the study. Single-cell RNA-sequencing were applied to analyze the immune cell composition of TNBC tumors and peripheral blood from patients receiving chemotherapy combined with ICIs. This study characterized the CD8Teff in evaluation of tumor microenvironment hot and cold. Furthermore, we investigated the interactions between CD8Teff cells and other immune and stromal cells within the TME, which may guide the development of personalized treatment strategies for TNBC

Although CD8T IHC scores are widely used to differentiate between "hot" and "cold" tumors, this approach may not fully reflect the complexity of the immune microenvironment. In some cases, patients with high CD8T expression still exhibit poor responses to immunotherapy, suggesting significant heterogeneity in CD8T subtypes and differing functional roles between responders and no-responders (Fig. S1). This study employs single-cell RNA sequencing (scRNA-seq) and integrative multi-omics to achieve a more precise distinction between "hot" and "cold" tumors (Fig. 1), which included four patients treated with ICIs between January 2021 and September 2021.

**Fig. 2:**
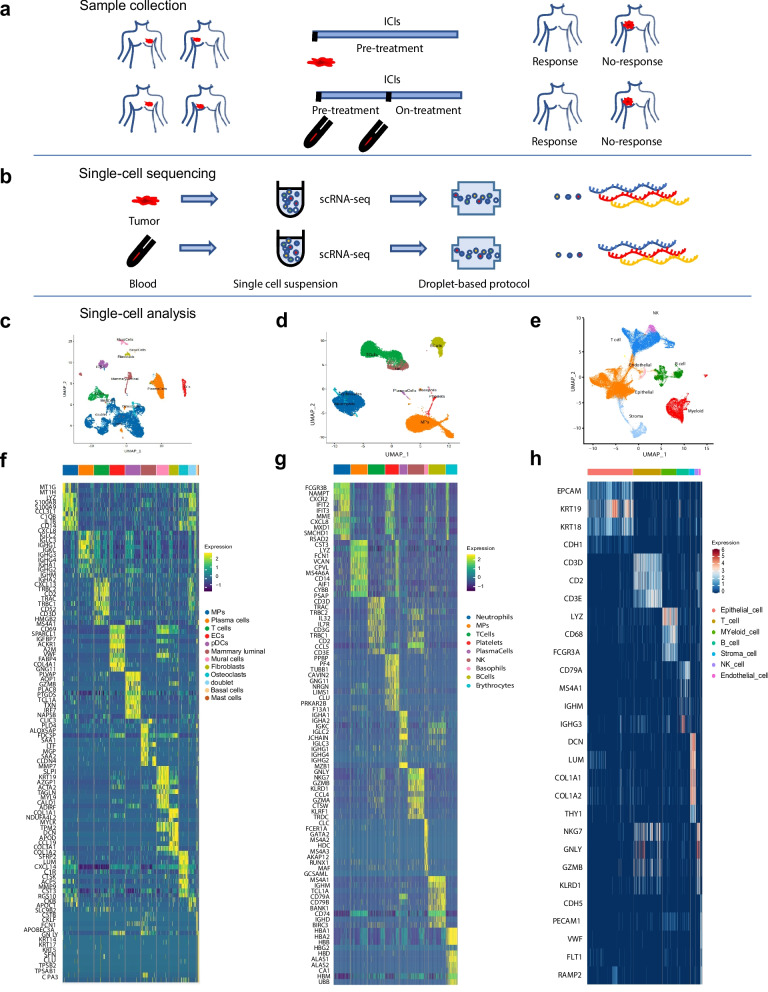
Immune cell dynamics in TNBC patients treated with ICIs. **a** Schematic of tissue and peripheral blood sample collection and (**b**) scRNA-seq workflow. **c** UMAP of scRNA-seq in all tumor tissue samples. **d** UMAP of scRNA-seq in all peripheral blood samples. **e** UMAP of scRNA-seq from 18 TNBC patients in GSE176078 and GSE161892 cohorts. **f** Heatmap representing gene expression profiles of immune cells in tumor tissue samples. **g** Heatmap representing gene expression profiles of immune cells in peripheral blood samples. **h** Heatmap showing the gene expression patterns of immune cells across 18 TNBC patients from the GSE176078 and GSE161892 cohorts

After four cycles of ICIs, two patients showed response, while the others did not (Fig. 2a, b). Single-cell RNA sequencing of two tumor tissues samples revealed distinct immune cell landscapes, including subclusters of T cells, B cells, NK cells, and stromal cells (Fig. 2c, f). The response exhibited significantly higher levels of Tfh and CD8Teff cells, while the no-response had more NKT and Treg cells (Fig. S2 a-c). These findings suggest that immune cell dynamics may provide insights into their functional importance in tumor immunity and the response to immunotherapy.

Further analysis of immune cell dynamics was conducted on peripheral blood samples collected from two additional advanced breast cancer patients before and after their first ICI treatment (Fig. 2d, g). T cells were classified into CD8Teff, NKT, naive T cells, Treg cells and GDT cells based on classical activation gene expression profiles (Fig. S2d-f). The results indicated dynamic shifts in T cell populations pre- and post-immunotherapy, with a marked increase in CD8Teff and NKT cells following treatment, signifying the activation of cytotoxic immune responses. Conversely, naive T cells decreased in proportion, suggesting their differentiation into more specialized subsets in response to therapy. These findings were consistent with broader analyses from 18 additional TNBC patients in the GSE176078 and GSE161892 cohorts, where similar immune cell distribution patterns were observed (Fig. 2e-h, Fig. S2g-i), further emphasizing the critical roles of CD8Teff and NKT cells in mediating immune responses to immunotherapy.

### Distinct CD8Teff pathways and immune cell interactions drive ICIs response

**Fig. 3:**
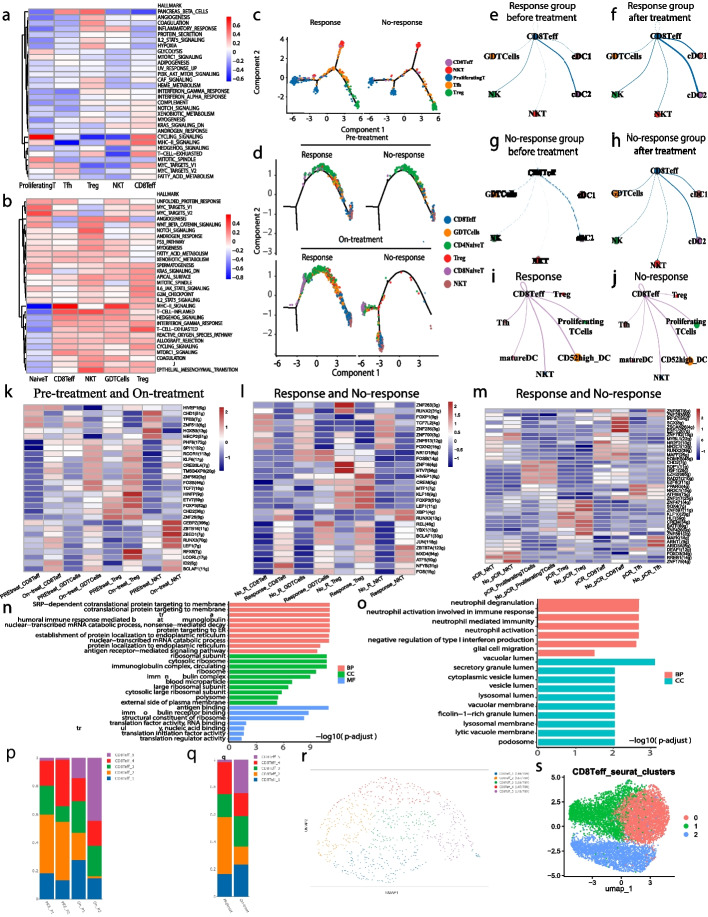
CD8Teff cells are the most functional T cell subsets in TNBC. **a** GSVA analysis heatmaps showing hallmark pathway enrichment in T cell subsets from tumor and (**b**) peripheral blood samples. **c** Pseudotime trajectory analysis of T cell subsets in tumor and (**d**) peripheral blood samples. **e**–**f** CellChat analysis of CD8Teff cell communication networks with other cells in the Response group (peripheral blood samples) before (**e**) and after (**f**) treatment. **g**–**h** CellChat analysis of CD8Teff cell communication networks with other cells in the No-Response group (peripheral blood samples) before (**g**) and after (**h**) treatment. **i**–**j** CellChat analysis of CD8Teff cell communication networks with other cells in the (**i**) Response group (tumor tissue samples) and (**j**) No-Response group (tumor tissue samples). **k** Heatmaps showing transcription factor in peripheral blood samples compared pre-treatment and on-treatment timepoints. **l** Heatmaps showing transcription factor in peripheral blood samples compared the Response group and No-Response group. **m** Heatmap of gene expression in tumor tissue samples from response and no-response groups. **n**–**o** GO enrichment analysis associated with CD8Teff cells in (**n**) peripheral blood samples and (**o**) 18 TNBC tissue samples derived from the GSE176078 and GSE161892 cohorts. **p**-**q** Stacked bar plots showing the distribution of CD8Teff cell subpopulations before and after treatment from peripheral blood samples. **r**-**s** UMAP clustering of CD8Teff cells based on Seurat analysis from (**r**) peripheral blood samples and (**s**) 18 TNBC tissue samples derived from the GSE176078 and GSE161892 cohorts

To explore the differences in immune response to immune checkpoint inhabitors, we performed Gene Set Variation Analysis (GSVA) on the Hallmark Pathways. Our analysis revealed significant differences in the pathways between T cell subgroups. In the tumor tissues samples, (Fig. 3a) T cells exhibited enhanced cell cycle signaling, indicating active proliferation, while CD8Teff cells in peripheral blood samples showed increased activation of MHC-II and exhaustion pathways, which are indicative of immune reactivation and improved tumor clearance (Fig. 3b). Differentiation trajectory analysis further revealed distinct differentiation paths for CD8Teff and Tfh cells, with varying distributions of proliferating T cells at the beginning and end of differentiation (Fig. 3c, d).

In response, CD8Teff cells formed strong communication networks comparing with other immune cells, including cytotoxic and helper immune cells, both before (Fig. 3e) and after immunotherapy (Fig. 3f). This suggests that responders maintain a coordinated and active immune microenvironment that is enhanced following treatment. In contrast, no-response exhibited weaker communication networks, with fewer interactions between CD8Teff cells and other immune subsets both before (Fig. 3g) and after treatment (Fig. 3h). Similarly, immune cell interactions differ markedly between the response and no-response groups (Fig. 3i-j) in tumor tissues.

Differences in transcription factor profiles were observed between T-cell subsets (Fig. 3k, 3 m) in the response and no-response groups, with T cells in the response group exhibiting enhanced activation of CD8Teff and NKT cells. Transcription factor profiles of T cell subsets differed markedly (Fig. 3l) Pre-treatment and On-treatment, with On-treatment samples showing enhanced activation of CD8Teff cells.

We then performed Gene Ontology (GO) enrichment analysis to further elucidate the biological processes associated with CD8Teff cells. CD8Teff cells were significantly enriched in processes related to immune activation, including cytotoxicity, T cell activation, and antigen presentation in both (Fig. 3n) tumor tissue and (Fig. 3o)18 TNBC tissue samples. In summary, CD8Teff cells exhibit distinct immune cytotoxic functions and enhanced intercellular interaction capabilities in the context of immunotherapy.

To further characterize CD8Teff, we conducted a subpopulation analysis in both (Fig. 3p, q, r) peripheral blood samples and (Fig. 3s) 18 TNBC tissue samples. In peripheral blood, the distribution of CD8Teff subsets shifted following treatment, with (Fig. 3p-q) CD8Teff-2 predominating Pre-treatment and CD8Teff-5 predominating On-treatment. 

### CD8Teff infiltration defines "Hot" tumors and predicts better clinical outcomes

**Fig. 4:**
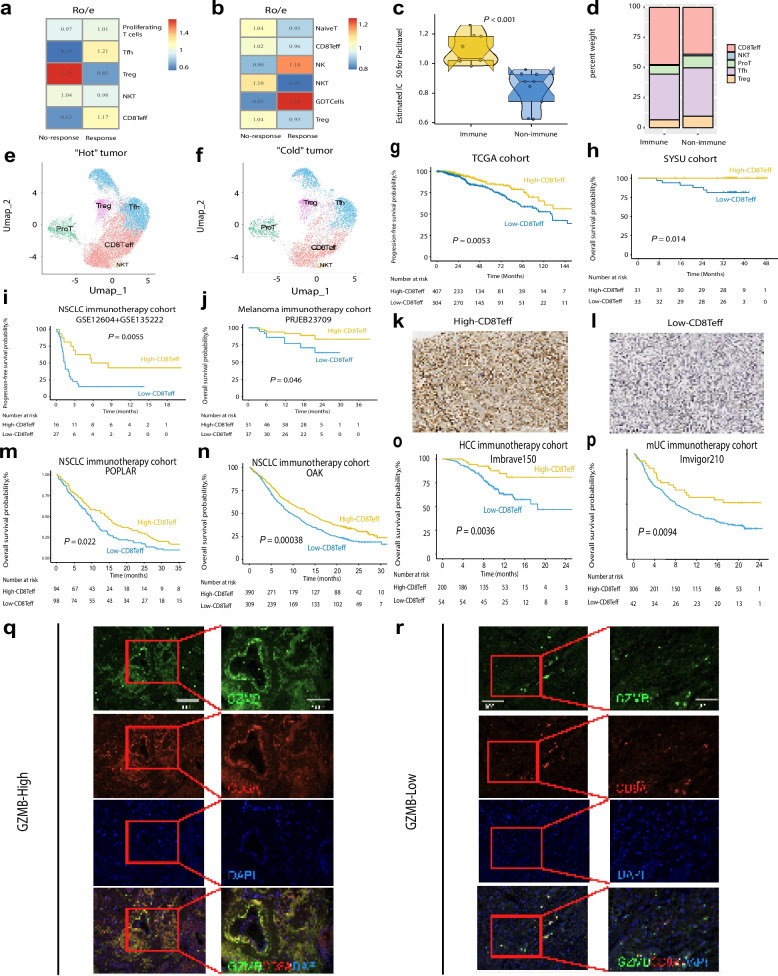
CD8Teff cells directly determine heat and cold of tumors. **a** Ro/e analysis of T cells in tumor tissues and (**b**) in peripheral blood based on single-cell sequencing data. **c** Box plot comparing CD8Teff cell proportions between "Hot" and "Cold" tumors by CD8Teff Ro/e in 18TNBC samples from GSE176078 and GSE161892 cohorts. **d** Bar plot showing the distribution of CD8Teff cells across different tumor response categories in 18TNBC samples fromGSE176078 and GSE161892 cohorts. **e** UMAP plots showing CD8Teff cell distribution in "Hot" and (**f**) "Cold" tumors. **g** Kaplan–Meier survival curves showing the association between CD8Teff cell levels and progression-free survival in TCGA cohort and (**h**) overall survival in SYSMH cohort. **i** Survival analysis of NSCLC immunotherapy cohort (GSE12604 + GSE135222) PFS based on CD8Teff levels. **j** Survival analysis of melanoma immunotherapy cohort (PRJEB23709) OS based on CD8Teff levels. IHC images showing GZMB expression in tumors with (**k**) high and (**l**) low CD8Teff infiltration. **m** Survival analysis of NSCLC immunotherapy cohort (POPLAR) OS based on CD8Teff levels. **n** Survival analysis of NSCLC immunotherapy cohort (OAK) OS based on CD8Teff levels. **o** Survival analysis of HCC immunotherapy cohort (Imbrave150) OS based on CD8Teff levels. **p** Survival analysis of mUC immunotherapy cohort (Immvigor210) OS based on CD8Teff levels. Immunofluorescence images illustrating (**q**) high and (**r**) low GZMB expression in tumor tissues

Ro/e scoring and tissue prevalence analysis confirmed significant differences in Tfh, Treg, and CD8Teff cells, underscoring their roles in driving immunotherapy response. The results indicated a significant difference in CD8Teff cell proportions between patients with different therapeutic responses. In tumor tissues, CD8Teff cells were more abundant in patients who responded to immunotherapy (Fig. 4a), while no-response exhibited higher proportions of Tregs and other immunosuppressive cells. A similar pattern was observed in peripheral blood, where CD8Teff cells were enriched in responders, highlighting their role in systemic immune activation (Fig. 4b).

Eighteen TNBC tumor samples were also classified based on Ro/e scores into "hot" and " cold" tumors."Hot"tumors, which displayed higher levels of CD8Teff cells, were more frequently found in responders, whereas"cold"tumors were enriched with Tregs (Fig. 4c-d). UMAP visualization further confirmed this differentiation, with "hot" tumors showing distinct clusters of CD8Teff cells, suggesting their active involvement in the anti-tumor immune response (Fig. 4e). In contrast,"cold"tumors displayed lower CD8Teff cell densities and a more heterogeneous immune profile, including higher proportions of Tregs and Tfh cells (Fig. 4f).

Kaplan–Meier survival analysis demonstrated that higher levels of CD8Teff cells were significantly associated with improved progression-free survival (PFS) (Fig. 4g) in patients from the TCGA cohort and overall survival (OS) (Fig. 4h) in patients from the Sun Yat-Sen Memorial Hospital, Sun Yat-Sen University (SYSMH) cohort. Patients with higher CD8Teff cell infiltration had better clinical outcomes, highlighting the importance of these cells in enhancing the effectiveness of immunotherapy. These results were further validated using IHC and external datasets (Fig. 4k-l). In the non-small cell lung cancer (NSCLC) ICIs cohorts (GSE126044 and GSE135222), higher CD8Teff levels were associated with improved PFS (Fig. 4i), while the melanoma ICIs (PRJEB23709) dataset confirmed a similar trend for OS (Fig. 4j). Similar results were observed across multiple immunotherapy cohorts, with high CD8Teff cell levels strongly associated with improved prognosis (Fig. 4. m, n, o, p). These findings emphasize the pivotal role of CD8Teff cells in fostering an immune-active tumor microenvironment and directly determining the "hot" or "cold" status of tumors, which is crucial for predicting therapeutic outcomes.

The gene expression analysis revealed that CD8Teff cells from responders showed upregulation of key cytotoxic genes, such as GZMB and PRF1, compared to non-responders (Fig. S3a). GO enrichment analysis highlighted that CD8Teff cells from responders were more active in immune-related processes, particularly in antigen processing and presentation pathways (Fig. S3b). Comparing pre-treatment and on-treatment samples, on-treatment CD8Teff cells exhibited higher expression of immune activation markers, indicating increased tumor-killing activity (Fig. S3c). GO analysis further supported this by showing enhanced immune activity and protein translation in on-treatment CD8Teff cells (Fig. S3d). Transcription factor analysis revealed that CD8Teff cells in responders expressed higher levels of pro-inflammatory cytokines and effector molecules, consistent with their active role in driving anti-tumor immunity (Fig. S3e, f).

To further validate these findings, immunohistochemical (IHC) staining for granzyme B (GZMB), a marker of cytotoxic activity, was performed. Tumors with high CD8Teff infiltration showed strong GZMB expression, while low CD8Teff infiltration tumors exhibited weak or absent staining (Fig. 4k, l). Immunofluorescence (IF) staining revealed similar results (Fig. 4q, r). Importantly, GZMB staining was more effective than CD8A staining in distinguishing "hot" from "cold" tumors, particularly in cases where CD8A staining was insufficient (Fig. 4q, r). This highlights GZMB’s utility in accurately distinguishing between hot and cold tumors, compensating for the limitations of traditional CD8A-based methods.

### CXCL13 correlates with CD8Teff activity and modulates tumor immune microenvironment

**Fig. 5:**
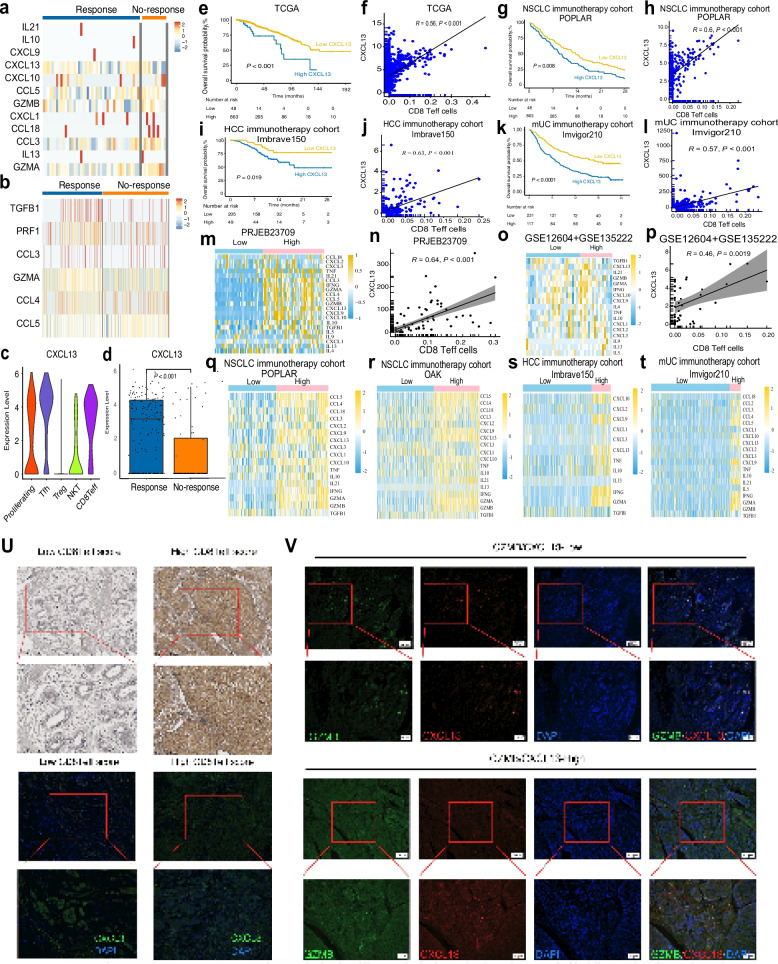
CXCL13 was the most distinct cytokine in CD8Teff cells. Heatmaps showing CXCL13 expression between response and no-response groups in tissues samples (**a**) and (**b**) peripheral blood samples. **c** Violin plots and box plots comparing CXCL13 expression between subgroups. **d** Box plot showing CXCL13 expression differences between response and no-response groups. **e**, **g**, **i**, **k** Kaplan–Meier survival analysis of CXCL13 expression in four immunotherapy cohorts: (**e**) TCGA, (**g**) POPLAR, (**i**) Imbrave150, and (**k**) Imvigor210. **f**, **h**, **j**, **l** Correlation of CXCL13 expression with CD8Teff cells in four immunotherapy cohorts: (**f**) TCGA, (**h**) POPLAR, (**j**) Imbrave150, and (**l**) Imvigor210. **m** Heatmap and (**n**) correlation analysis of CXCL13 in the melanoma ICIs dataset (PRJEB23709). **o** Heatmap and (**p**) correlation analysis of CXCL13 in the NSCLC ICIs datasets (GSE12604 + GSE135222). **q**–**t** Heatmaps of immune-related cytokine expression between CD8Teff high vs. low groups across four immunotherapy cohorts: (**q**) POPLAR, (**r**) OAK, (**s**) Imbrave150, (**t**) Imvigor 210. **u** IHC and IF images showing CXCL13 expression in CD8Teff-low and CD8Teff-high scoring tumors. **v** IF images showing GZMB expression in CXCL13-low and CXCL13-high scoring tumors

To further investigate the role of the tumor microenvironment, we analyzed cytokine profiles in both blood and tissue samples across different groups. Significant cytokine variations were detected between these groups, with CXCL13 emerging as a critical cytokine that potentially affects multiple pathways in tumor microenvironment (Fig. 5 a, b). CXCL13 was differentially expressed in various T cell subpopulations and response groups, with statistically significant differences (Fig. 5 c, d). TCGA cohort confirmed that CXCL13 influences cell survival and is closely correlated with CD8Teff activity, reinforcing its role as a key modulator in the tumor microenvironment (Fig. 5e,). CXCL13 expression is significantly positively correlated with CD8⁺ Teff cell infiltration, and samples with high CXCL13 expression are broadly enriched in immune-related genes (Fig. 5f). Consistent results were observed in NSCLC ICIs cohorts, HCC immunotherapy cohort, mUC immunotherapy cohort (Fig. 5g-l). To further validate the differences in cytokine expression profiles driven by CD8⁺ Teff cells, we analyzed additional immunotherapy cohorts. Across the melanoma immunotherapy cohort (PRJEB23709), the NSCLC ICIs cohorts (GSE126044, GSE135222, POPLAR, and OAK), the HCC immunotherapy cohort (Imbrave150), and the mUC immunotherapy cohort (IMvigor 210), CXCL13 consistently emerged as a cytokine whose expression differed significantly under CD8⁺ Teff cell–driven conditions (Fig. 5m-t)n addition, immunohistochemistry (IHC) and immunofluorescence (IF) analyses revealed distinct spatial distributions of CXCL13 corresponding to different GZMB expression states, further supporting the co-expression relationship between GZMB and CXCL13 (Fig. 5u-v). These findings highlight the significant role of CXCL13 in modulating immune responses and its close association with CD8Teff cell activity within the tumor microenvironment.

### CD8Teff coordinate TME remodeling and metabolic reprogramming

**Fig. 6:**
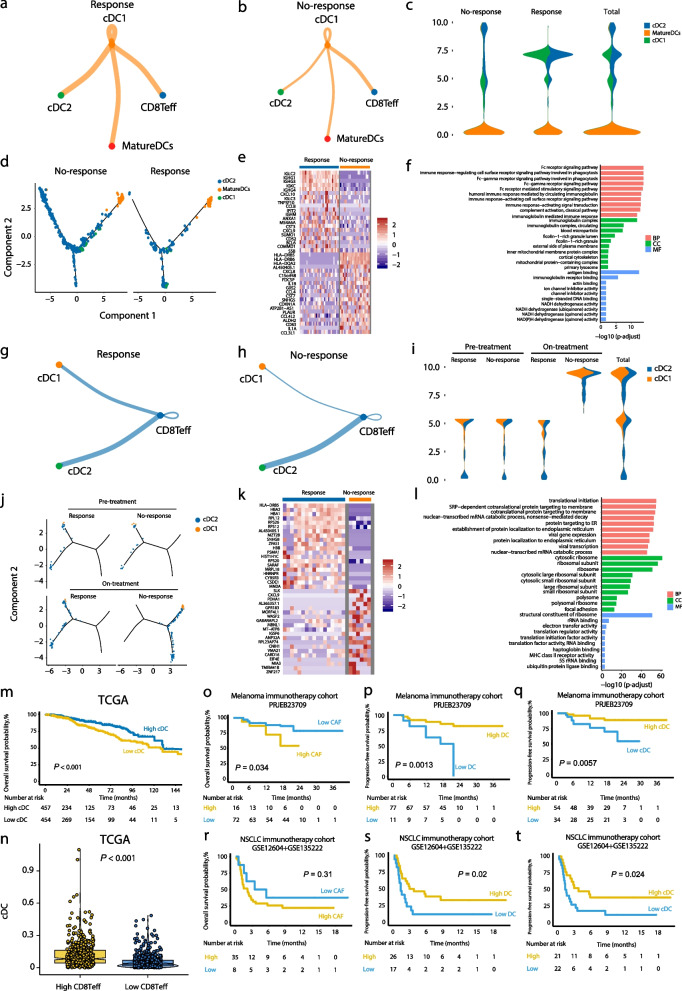
CD8Teff cells affect antigen presentation and differentiation in DC cells. **a** Cell–cell communication network in response and (**b**) no-response groups. **c** Proportion of DC subsets between different groups in tumor samples. **d** Pseudotime trajectory analysis showing differentiation paths of DCs in no-response and response groups. **e** Heatmap of gene expression in DCs from no-response and response groups. **f** GO enrichment analysis highlighting biological processes involved in DC function in the response group. Cell–cell communication network in (**g**) response and (**h**) non-response. **i** Proportion of DC subsets in different groups in blood samples. **j** Pseudotime trajectory analysis showing differentiation paths of DCs at different treatment stages. **k** Heatmap of gene expression in DCs from response and no-response. **l** GO enrichment analysis highlighting biological processes involved in DC function in responder groups. **n** Box plots comparing the expression of CD8Teff cells between high-DC and low-DC groups in TCGA cohort. Kaplan–Meier survival analysis based on CAF expression levels in (**o**) PRJEB23709 and (**r**) NSCLC(GSE12604 + GSE135222); Kaplan–Meier survival analysis based on cDC expression levels in these cohorts: (**m**) TCGA, (**q**) PRJEB23709, and (**t**) NSCLC(GSE12604 + GSE135222). Kaplan–Meier survival analysis based on DC expression levels in these cohorts: (**p**) PRJEB23709 and (s) NSCLC(GSE12604 + GSE135222)

Given the critical function of CD8Teff cells in antigen recognition and immune activation, we explored their interaction with dendritic cells. Single-cell analysis revealed distinct subsets of DCs, including cDC1, cDC2, and mature DCs, with a stronger association between CD8Teff cells and cDC1 in the response group from tumor tissues samples (Fig. S4, Fig. 6a, b). Trajectory analysis showed that cDC1 cells were more prevalent in the response group (Fig. 6c, d). Gene expression analysis revealed upregulation of antigen presentation-related genes in cDC1, particularly those involved in immune signaling and Fc receptor activity (Fig. 6e, f). Cell–chat analysis revealed that CD8Teff exhibited stronger interactions with conventional dendritic cell subsets cDC1 and cDC2 in the response group from peripheral blood samples (Fig. 6g-h). Pseudotime trajectory analysis revealed distinct temporal differentiation patterns of cDC1 and cDC2 subsets between immunotherapy-responsive and non-responsive groups (Fig. 6i-j). Additionally, response patients showed increased activity in protein translation and ribosomal processes (Fig. 6k, l). Kaplan–Meier survival analysis indicated that higher DC infiltration was significantly associated with better survival outcomes (*P* = 0.007) (Fig. 6m), and the presence of CD8Teff cells in DC-rich tumors further highlighted the role of cDC1 in enhancing anti-tumor immune responses (Fig. 6n). This supports the crucial role of cDC1 in presenting antigens to CD8Teff cells, enabling tumor-specific killing.

Further analysis showed that high levels of CAFs were associated with worse survival, suggesting that CAFs contribute to a more suppressive tumor microenvironment (Fig. 6o, r). In contrast, higher infiltration of DC and cDC cells was consistently linked to improved survival, with significant results observed in both the melanoma ICIs cohort (*P* = 0.0013 for DCs, *P* = 0.0057 for cDCs) and the NSCLC ICIs datasets (*P* = 0.02 for DCs,* P* = 0.024 for cDCs) (Fig. 6p, q, s, t). These findings highlight the role of high CAF levels in promoting immune suppression, while elevated DC infiltration supports a more active immune response, thereby improving patient prognosis. In summary, CD8Teff cells play a central role in shaping the tumor microenvironment by influencing tumor cells, fibroblasts, and endothelial cells. Their interaction with various cell types highlights their importance in modulating both tumor progression and immune responses.

We further investigated how CD52 + dendritic cells (DCs) regulate CD8Teff cell activity. CD52 + DC populations were enriched in response samples, indicating their active role in promoting more effective immune responses (Fig. S5a). Pathway analysis showed strong connections between CD52 + DCs and CD8Teff cells, with enhanced antigen presentation and immune activation in responders (Fig. S5b, c). CD52 + DCs exhibited upregulation of genes linked to immune activation, such as TNFSF10, CCL5, and CCL22, in response samples, while non-responders showed weaker expression patterns (Fig. S5d, e). Furthermore, response patients demonstrated enhanced signaling in immune-regulating pathways, such as TNF and CXCL, indicating that CD52 + DCs play a key role in modulating immune responses and shaping the tumor microenvironment (Fig. S5f, g). Thus, CD52 + DC cells regulate CD8Teff activity by modulating antigen presentation and directly influencing cytokine activity, ultimately shaping the tumor microenvironment.

**Fig. 7:**
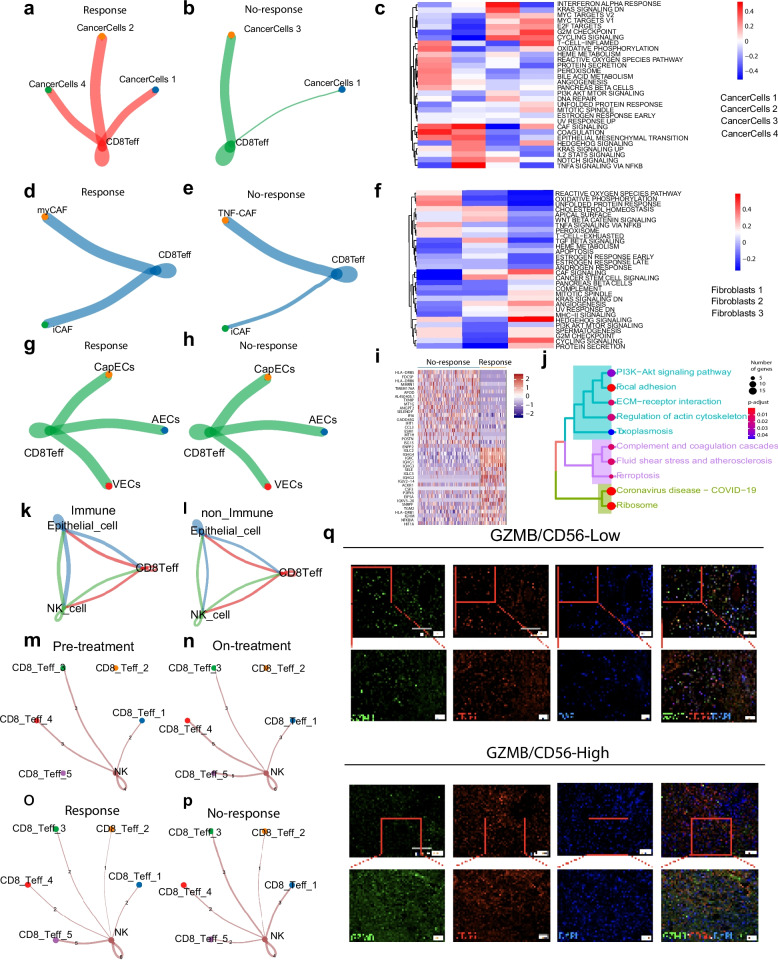
CD8Teff cells affect the function of tumour cells, fibroblasts and endothelial cells. Network diagrams representing the interactions between CD8Teff cells and different tumor cell subtypes in (**a**) response and (**b**) no-response groups. **c** Heatmap of hallmark pathways affected by CD8Teff interactions with various cancer cell subtypes. Network diagrams of CD8Teff cell interactions with fibroblast subtypes in (**d**) response and (**e**) no-response groups. **f** Heatmap of hallmark pathways affected by CD8Teff interactions with various fibroblasts subtypes. Network diagrams of CD8Teff cell interactions with endothelial cell subtypes in (**g**) response and (**h**) no-response groups. **i** Heatmap of gene expression in the endothelial cells. **j** Enriched pathways impacted by CD8Teff cells across different groups. Network diagrams representing the interactions between CD8Teff cells, epithelial cell and NK cell in (**k**) response and (**l**) no-response groups. **m**, **n**, **o**, **p** Network diagrams representing the interactions between different tumor cell subtypes of CD8Teff cells in (**m**) pre-treatment, (**n**) on-treatment and (**o**) response, (**p**) no-response groups. **q** Immunofluorescence co-expression of GZMB and CD56 under high and low expression conditions

Building on these findings, we extended our analysis to other key components of the tumor microenvironment, such as tumor cells, fibroblasts, and endothelial cells (Fig.S6). CD8Teff cells exhibit strong communication with CancerCells_2 and CancerCells_4 (Fig. 7 a, b), which are associated with the upregulation of interferon-alpha response, apoptosis, and epithelial-mesenchymal transition (EMT) pathways, further supporting their potential role in tumor suppression and remodeling of the tumor microenvironment (Fig. 7 a, b, c). Additionally, CD8Teff cells exhibited particularly strong interactions with myCAF (Fig. 7 d, e), activating pathways such as TNF signaling and cytokine-cytokine receptor interactions, which may contribute to immunomodulatory functions in the tumor microenvironment (Fig. 7d, e, f). In contrast, low CD8Teff expression was linked to stronger interactions between tumor cells and microvascular endothelial cells (Fig. 7g, h), with ECM pathway activation being more prominent in no-response, indicating a potential link to increased metastatic potential (Fig. 7i, j). Stronger communication has shown between epithelial cell, CD8Teff and NKT cells in the response group (Fig. 7k, l). The frequency and intensity of CD8Teff–NK cell interactions between response and no-response groups is different (Fig. 7k, l). The CD8Teff-5 subpopulation exhibited stronger associations with NK cells on-treatment (Fig. 7m, n). Notably, in the response group, CD8⁺ Teff-5 demonstrated particularly close interactions with NK cells (Fig. 7o, p).

To further investigate the relationship between CD8Teff and NK cells, we performed spatial co-localisation analysis using multiplex immunofluorescence, which demonstrated similar expression abundances and spatial distributions for CD8Teff and NK cells (Fig. 7q).

Additionally, pathway enrichment analysis revealed that key metabolic processes, such as glycolysis, fatty acid oxidation (FAO), and glutaminolysis, were significantly upregulated in response patients, highlighting the importance of these metabolic pathways for effective anti-tumor responses (Fig. S7a, b). In the response group, CD8Teff cells primarily exhibited enhanced amino acid metabolism and lipid biosynthesis, supporting their activated and functional state. In contrast, CD8Teff cells from the non-responsive group displayed a metabolism dominated by glucose utilization, with elevated glycolytic activity suggesting a shift toward functional exhaustion (Fig. S7c). Patients with high CD8Teff infiltration also showed increased activity in glycolysis, FAO, and the pentose phosphate pathway (PPP), linking CD8Teff cells to metabolic reprogramming within the tumor (Fig. S7d-i). These findings suggest that metabolic shifts driven by CD8Teff cells are critical for shaping the tumor microenvironment and enhancing anti-tumor immunity.

### AI-based spatial mapping of CD8Teff enables precision stratification

**Fig. 8:**
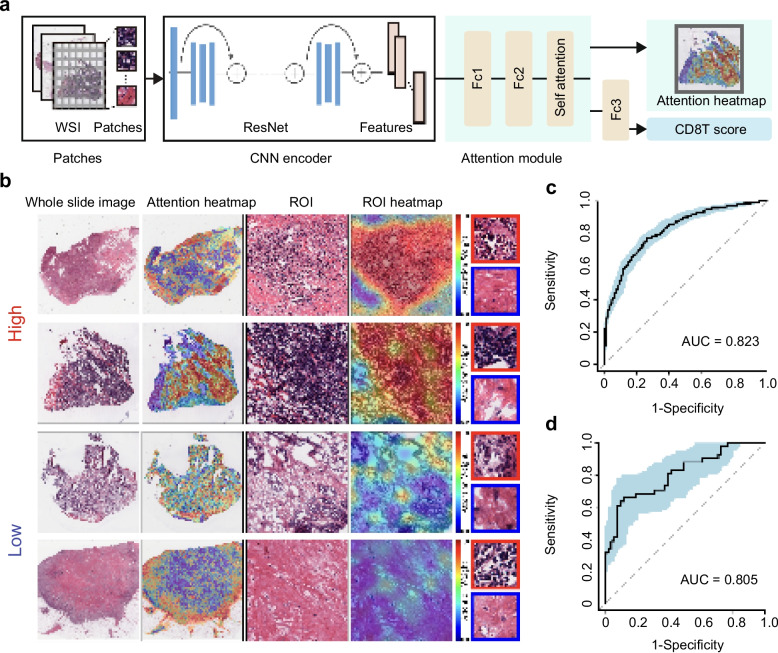
Pathological AI detects CD8Teff expression and spatial heterogeneity. **a** design process of the CD8Teff pathology artificial intelligence (AI) algorithm, (**b**) visualizing CD8Teff distribution heterogeneity in pathology slices, the AUC of pathology based-AI identified CD8Teff is (**c**) 0.823 in the training group and (**d**) 0.805 in the validation group

To assist clinical decision-making and enhance the understanding of tumor immunity, we developed a network of AI algorithms using pathological images of TNBC patients from the TCGA database, aimed at identifying CD8Teff within the tumor microenvironment as key determinants of "cold" and "hot" tumor classification. A total of 836 HE pathology slices from the TCGA public database were analyzed, quantifying immune cell distributions with high specificity (Fig. 8a). Pathologically identified CD8Teff-enriched regions from patients with high CD8Teff expression and CD8Teff-sparse regions from patients with low CD8Teff expression were highlighted. Analysis revealed significant spatial heterogeneity in CD8Teff distribution (Fig. 8b), underscoring the variability in immune infiltration within the tumor microenvironment. This spatial heterogeneity provides a foundation for interpreting immune cell distribution in pathology images and sets the stage for the future quantification of additional immune markers. The reliability of CD8Teff identification was validated, achieving an AUC of 0.823 in the training group and 0.805 in the validation group, confirming the accuracy of AI-driven immune cell classification (Fig. 8c, d).

This AI-driven analysis plays a crucial role in clinically distinguishing between "hot" and "cold" tumors, aiding in personalized treatment strategies. Patients with "cold" tumors, characterized by low CD8Teff activity, may benefit from anti-angiogenic therapies that target the tumor-supportive microvascular environment. In contrast, patients with "hot" tumors, which exhibit higher CD8Teff infiltration, typically respond better to immunotherapy combined with chemotherapy. By aligning treatments with the tumor immune profile, this precision-driven approach not only optimizes therapeutic outcomes but also represents a significant advancement in personalized cancer care.

### CD8Teff cells drive tumor immunity status and inform personalized therapy

CD8Teff cells serve as a critical marker for distinguishing tumor immunotypes and provide essential insights for guiding personalized treatment. These cells modulate the tumor microenvironment by influencing DC antigen presentation, which enhances CD8Teff cell activation. In addition, CD8Teff cells are linked to the expression of CXCL13, a key cytokine that helps regulate immune responses and further amplifies CD8Teff cell activity. Together, this process drives changes in the tumor microenvironment, affecting the differentiation of tumor cells, CAFs, and endothelial cells. In "hot" tumors, CD8Teff cells are more active, creating an immune-responsive environment that is more receptive to immunotherapy (Fig.S8).

For clinical applications, this distinction between "hot" and "cold" tumors is vital. Patients with "cold" tumors, characterized by low CD8Teff activity, may benefit from anti-angiogenic therapies that target the supportive microvascular environment promoting tumor growth. In contrast, patients with "hot" tumors, which have higher CD8Teff infiltration, often achieve better outcomes with immunotherapy combined with chemotherapy, enhancing the immune system's ability to fight the tumor. This personalized treatment approach can significantly improve therapeutic effectiveness depending on the tumor's immune profile.

## Discussion

In this study, single-cell RNA sequencing analyses of tumor tissues and peripheral blood from TNBC patients undergoing ICIs were conducted to investigate factors associated with the ICIs treatment efficacy. This study provides crucial insights into the immune landscape of TNBC, revealing distinct immune cell dynamics in response to chemotherapy combined with ICIs. Through scRNA-seq, the study identified significant differences in the composition and functionality of immune cell subsets between Response patients and No-response patients. Notably, CD8Teff cells emerged as a key determinant of immunotherapy efficacy and tumor classification as "hot" or "cold", and CD52 + DCs modulate CD8Teff cell activity by influencing antigen presentation. Furthermore, activated CD8Teff cells enhance their tumor-killing capacity by secreting increased levels of CXCL13, which mediates interactions with NK cells, tumor cells, CAFs, and vascular endothelial cells. Which interactions play a critical role in shaping the tumor microenvironment and ultimately affect the efficacy of immunotherapy. These findings offer clinicians enhanced tools for assessing the likely success of immunotherapy in breast cancer patients, ultimately contributing to more personalized treatment strategies. Furthermore, the study guides clinicians in developing treatment regimens tailored to differential CD8Teff expression to enhance therapeutic efficacy and employs AI-based pathological models to directly identify and quantify CD8Teff cells, thereby supporting clinical decision-making in immunotherapy. This study addressed the challenge of efficiently and directly identifying the state of the tumor immune microenvironment and elucidated its underlying mechanisms. By characterizing CD8Teff status, we guided the selection of immunotherapy strategies tailored to individual patients and implemented a pathological AI model capable of directly identifying CD8Teff cells, thereby facilitating clinical translation. This approach supports precision treatment and aims to enhance the efficacy of immunotherapy in TNBC.

CD8Teff cells are pivotal in defining the "hot" or "cold" status of the TME and possess significant potential serve as clinical diagnostic biomarkers for guiding immunotherapy decisions. The study demonstrated the robust strong capability of CD8Teff cells to distinguish between "hot" and "cold" TME through integrated single-cell and multi-transcriptome data analysis. This approach challenges traditional methods that rely on multifactorial indicators such as CD8 + T cell staining, tumor mutation burden, or active cytokines, offering a more direct and precise means of categorizing tumor immune activity. By providing clinicians and pathologists with a more convenient assessment index, it reduces the diagnostic complexity and workload for pathologists, thereby improving the diagnostic efficiency for patients with TNBC. Furthermore, efficient AI-enabled pathological recognition of CD8Teff cells facilitate the classification of tumors as "cold" or "hot", presenting a novel approach to advancing tumor diagnosis and prognosis.

Different adjuvant and neoadjuvant treatment regimens may be recommended for TNBC patients based on their different CD8Teff typing. Clinical evidence from trials such as POPLAR, OAK, IMbrave150, and IMvigor210 has demonstrated that "hot" tumors, characterized by higher CD8Teff levels, exhibited stronger immune activation and improved clinical outcomes [10]. In contrast, the"cold"tumors, which are enriched with immunosuppressive cells like Tregs, show poorer responses to immunotherapy and are correlated with shorter PFS and OS.

Immunotherapy-based combination regimens can be tailored according to CD8Teff phenotypes and their associated tumor microenvironment molecular signatures. The FUTURE-C-PLUS/FUTURE-SUPER study found that the combination of chemotherapy and immunotherapy with anti-angiogenic drugs improved TME and TNBC achieving objective response rates of up to 77% [16, 17]. For patients with cold tumors and low CD8Teff expression, a treatment strategy incorporating immunotherapy and anti-angiogenic drugs is recommended to suppress tumor invasion and metastasis by enhancing anti-angiogenesis [18]. Additionally, administering active cytokines is advised to modulate the inert TME and increase the sensitivity of TNBC patients to immunotherapy. KEYNOTE-522 [5, 19], KEYNOTE-355 [3, 20] and TORCHLIGHT [21] have demonstrated that both early-stage and advanced TNBC can benefit from immunotherapy, especially when combined with albumin-paclitaxel. For patients with "hot" tumors and high CD8Teff expression, immunotherapy combined with albumin-paclitaxel-based chemotherapy is the preferred approach remains the preferred therapeutic approach.

CD8Teff cells hold significant potential as biomarkers for predicting immunotherapy response, and enhanced CD8Teff activity may transform "cold" tumors into "hot" tumors, fostering a more immune-responsive microenvironment and ultimately improving patient prognosis. The OXEL study demonstrated that both peripheral immune cell scores and circulating tumor DNA status were strongly associated with an increased risk of relapse in patients with early TNBC [22]. Similarly, the KEYNOTE-355 study demonstrated that patients with PD-L1 combined positive score (CPS) ≥ 10 experienced improved PFS and OS [23]. However, the predictive capacity of PD-L1 scores remains limited, as they do not consistently reflect the efficacy of immunotherapy [24]. In contrast, this study offered a more direct and reliable indicator of tumor immune status and therapy response by utilizing CD8Teff scores. Unlike with PD-L1 scores, the CD8Teff score integrates multiple complex factors within the TME, including interactions with DCs, CAFs, cancer cells, NK cells and cytokines, offering a more accurate characterization of tumor immune activity and therapy response. These elements contribute to a more accurate characterization of tumor immune activity and treatment response. Moreover, alterations in pathways such as MYC and KRAS play a critical role in converting tumors from a "cold" to a "hot" phenotype, further highlighting the importance of targeting these mechanisms to enhance the effectiveness of immunotherapy.

CD8Teff cells contribute to metabolic reprogramming of the tumor microenvironment, enhancing antitumor immune responses and modulating tumor progression. The strong correlation between CD8Teff cells and metabolic reprogramming within the tumor microenvironment suggests that CD8Teff-driven metabolic shifts, including upregulation of glycolysis and fatty acid oxidation, which are essential for sustaining effective anti-tumor responses [25–27]. These findings highlight the therapeutic potential of targeting metabolic pathways to enhance CD8Teff functionality and improve immunotherapy outcomes. Targeting metabolic pathways to enhance CD8Teff activity could be a promising approach to boost the effectiveness of immunotherapies [28]. Drugs or interventions that modulate key metabolic processes, such as glycolysis or fatty acid oxidation, could support the energy demands and functional persistence of CD8Teff cells within the metabolically challenging TME. potentially transforming the tumor microenvironment from a metabolically hostile one into a more supportive environment for immune-mediated tumor destruction. Enhancing glycolysis through upregulation of GLUT1 or promoting fatty acid oxidation via CPT1A activation can reinforce effector function and cytotoxicity. These interventions have the potential to remodel the TME from an immunosuppressive, nutrient-depleted state into one that is more conducive to sustained immune activation and tumor eradication.

CD8⁺Teff function is shaped by a complex network of stimulatory and inhibitory interactions within TME. DCs play a central role in priming naïve CD8⁺ T cells by presenting antigenic peptides via MHC-I and delivering co-stimulatory signals. In turn, activated CD8Teff cells secrete IFN-γ, which enhances DC maturation and antigen-presenting capacity, forming a positive feedback loop that amplifies anti-tumor immunity. NK cells also contribute to this network by supporting CD8⁺ T cell–mediated tumor clearance through IL-15–mediated cross-talk and complementary cytotoxic mechanisms. Conversely, CAFs exert immunosuppressive effects by producing extracellular matrix components that physically hinder immune cell infiltration. They also secrete cytokines such as TGF-β and IL-6, which promote immune evasion and tumor progression. CAFs further recruit and sustain Tregs and myeloid-derived suppressor cells (MDSCs), both of which inhibit CD8⁺ T cell function. A comprehensive understanding of these intercellular dynamics is essential for designing synergistic and precise therapeutic interventions to restore and potentiate CD8⁺Teff functionality.

Despite the valuable insights gained, the study still has the following shortcomings. First, there was inconsistency in the single-cell RNA sequencing platforms used—tissue and blood samples were sequenced using different technologies compared to the GEO public database datasets, which may introduce technical variability. Second, the study primarily focused on the characterization and mechanism of CD8Teff, and although the findings are compelling, they serve as supportive evidence for future basic mechanistic investigations. Third, the study did not include a prospective clinical cohort evaluating the effects of anti-angiogenic therapies. Future studies should incorporate prospective cohorts to explore the therapeutic efficacy and mechanistic contributions of combining anti-angiogenic agents with immunotherapy and chemotherapy, particularly in patients with'cold'tumors.

In summary, the study identifies CD8Teff as pivotal markers for immunotherapy response in TNBC. Through integrated single-cell RNA sequencing and AI-based analysis, high CD8Teff levels correlate with "hot" tumors characterized by active immune responses and favorable treatment outcomes, whereas "cold" tumors with low CD8Teff infiltration exhibit reduced responsiveness to ICIs. CD8Teff cells actively modulate through cytokine signaling and immune activation, underscoring their critical role in shaping treatment response. These findings support the implementation of CD8Teff-based stratification as a foundation for personalized immunotherapy strategies in TNBC.

## Materials and methods

### Study design and patients

This study prospectively enrolled four TNBC patients from Sun Yat-Sen Memorial Hospital, Sun Yat-Sen University (China) between January and September 2021. Two early-stage TNBC patients received neoadjuvant chemotherapy combined with immune checkpoint inhibitors (ICIs); one achieved a pathological complete response (pCR, defined as the response), and the other did not (non-pCR, defined as the no-response). Additionally, two advanced TNBC patients receiving first-line chemoimmunotherapy were included, with blood samples collected before and after treatment; one response and the other did not (Supplementary Table 1).

Publicly available single-cell transcriptomic data from TNBC patients (GSE176078, GSE161892) were incorporated for validation (Supplementary Table 2). Bulk RNA-seq data from the TCGA-BRCA cohort and additional immunotherapy datasets were also analyzed, including: Melanoma: PRJEB23709 (European Nucleotide Archive); NSCLC: GSE126044, GSE135222 (GEO), POPLAR (NCT01903993), and OAK (NCT02008227); HCC: Imbrave150 (NCT03434379); mUC: IMvigor210 (NCT02951767, via IMvigor210CoreBiologies R package). Samples with missing clinical or survival data were excluded. All datasets included transcriptomic profiles, immunotherapy response classifications, and survival outcomes (PFS/OS) (Supplementary Table 2). Above mentioned datasets were accessed and downloaded in September 2021 and have remained publicly available since then.

### Single-cell transcriptome library preparation and sequencing

Fresh tumor tissues were preserved in sCelLive™ solution (Singleron Bio), processed within 30 min post-resection. Samples were minced, enzymatically dissociated using GEXSCOPE™ solution, filtered, and centrifuged. Erythrocytes were removed, and viable cells were counted with trypan blue. Cell suspensions (1 × 10^5^ cells/mL) were loaded onto microfluidic chips for library construction using the GEXSCOPE™ Single-Cell RNA Library Kit and sequenced on an Illumina NovaSeq 6000 (150 bp paired-end reads).

Reads were processed with CeleScope v1.1.7. Cutadapt v1.17 was used for trimming [29], STAR v2.6.1a for genome alignment (GRCh38) [30], and featureCounts v2.0.1 for UMI quantification [31]. Cells were excluded if they had < 200 or top 2% of gene counts, > 50% mitochondrial reads, or genes expressed in < 5 cells. After filtering, 13,334 high-quality cells were retained (avg. 1,869.6 genes and 7,975.4 UMIs/cell).

Seurat v3.1.2 was used for normalization, scaling, PCA, and clustering (resolution = 2.0, 30 clusters). UMAP was used for 2D visualization. DEGs were identified with Seurat’s FindMarkers (Wilcoxon test, logFC > 0.25, expression in > 10% of cells). Cell types were annotated using canonical markers and the SynEcoSys database (Supplementary Tables 2–3). Doublets were excluded based on co-expression of multiple lineage markers.

Pathway and trajectory analysis. Pathway enrichment analysis was conducted using clusterProfiler v4.0.2 [32] for GO and KEGG, considering pathways with p_adj < 0.05 as significant. Gene set variation analysis (GSVA) was performed using the GSVA package v1.44.2 [33] to examine pathway differences across cell types.

For cell differentiation trajectory analysis, Monocle2 v2.22.0 [34] was used with highly variable genes to order cells by differentiation stage. Dimension reduction was achieved with DDRTree, and key differentiation paths were identified.

Transcription factor network analysis. Transcription factor regulatory networks were inferred using pyscenic v0.11.0 [35], starting with scRNA expression data and transcription factors from AnimalTFDB. Networks were predicted with GRNBoost2, refined with CisTarget to identify transcription factor binding motifs, and visualized using pheatmap in R.

Macrophage gene modules were detected using Hotspot [36] (top 500 genes with highest autocorrelation z-scores). Modules were scored and Jaccard similarity coefficients were calculated to assess transcriptional similarity between immune subtypes [37].

CellPhoneDB v2.1.7 was used to infer ligand–receptor interactions (p < 0.05, log-expression > 0.1) [38]. Circlize v0.4.10 (R) was used for visualization.

### Transcriptome sequencing data analysis

The xCELL algorithm, based on the ssGSEA method and gene set markers, was used to estimate the abundance of 64 cell types across five major categories: adaptive and innate immune cells, hematopoietic progenitor cells, epithelial cells, and extracellular matrix cells (including non-immune cell types). Following the GitHub protocol, we performed calculations using the functions *rawEnrichmentAnalysis*, *transformScores*, and *spillOver*. This process yielded results for 64 cell subtypes per sample, along with ImmuneScore, StromaScore, and MicroenvironmentScore. Kaplan–Meier survival curves were generated using the *survival* package in R.

Normalization and identification of differentially expressed genes (DEGs) were performed using the *DESeq* package in R. DESeq applies a negative binomial distribution (NB) test to assess differential read counts while correcting for length-related bias. Gene expression levels were estimated using the *baseMean* value. Genes with a log₂ fold change > 1 or <  − 1 and *p* < 0.05 were considered significantly differentially expressed. Pathway enrichment analysis, including Gene Ontology (GO) and KEGG, was performed using the *clusterProfiler* R package. GO terms and KEGG pathways with *p*-values and false discovery rates (FDR) < 0.05 were considered significant. GSEA was based on 1,000 permutations and FDR-adjusted *p*-values. Results were visualized using *enrichplot*. GSVA was conducted using gene sets from the MSigDB database.

### Multiplex immunofluorescence

FFPE tissue sections were deparaffinized twice with xylene and rehydrated through graded ethanol (95%, 85%, 75%), followed by rinsing with PBS. Sections were heated in citrate buffer (pH 6.0) at 100 °C for 30 min, cooled, and washed with PBS. They were incubated in 1% BSA for 30 min at room temperature to block non-specific binding. Sections were then incubated overnight at 4 °C with primary antibody (1:200 in BSA), followed by three 5-min PBS washes. HRP-conjugated secondary antibody (1:400, TSA kit) was applied for 60 min at room temperature, then washed three times with PBS. Endogenous peroxidase was blocked using 0.3% H₂O₂ for 10 min, followed by PBS washes. TSA-tyramine substrate with fluorescent dye (1:200) was added for 15 min at room temperature, followed by PBS washes. Antigen retrieval was repeated at 95 °C for 25 min, followed by PBS washes. These steps were repeated for additional antibody staining. Sections were mounted with antifade medium containing DAPI and sealed with coverslips. Stained sections were analyzed using a fluorescence microscope.

### Immunohistochemistry

Paraffin sections were baked at 70 °C for 50 min, then deparaffinized in xylene (15 min, twice) and rehydrated through a graded ethanol series (100% ethanol for 5 min, 95% for 5 min, 75% for 3 min). Sections were rinsed in PBS for 3 min (three times). Antigen retrieval was performed using sodium citrate buffer (pH 6.0) under high pressure for 30 min, followed by natural cooling and PBS rinses. To block endogenous peroxidase activity, 3% hydrogen peroxide was applied for 15 min at room temperature, followed by PBS rinses. Sections were blocked with goat serum for 30 min, and primary antibody was applied for overnight incubation at 4 °C. After rewarming, sections were incubated with secondary antibody (Zhongshan Jinqiao kit) for 30 min. DAB staining (1:20) was monitored microscopically, followed by hematoxylin counterstaining, dehydration, and mounting with neutral gum.

### Developing a pathological AI that recognizes CD8Teff expression and spatial heterogeneity

Each pathology slide was initially segmented into distinct regions to enable localized analysis of immune cell presence. Feature extraction was performed on these regions using a ResNet convolutional neural network (CNN), which captured intricate morphological characteristics associated with immune cell density. The extracted features were processed through a self-attention mechanism module followed by a fully connected layer, enabling binary classification of tumors as either CD8Teff-enriched or CD8Teff-sparse. This approach represents a key advance in quantifying immune cells from pathology images.

### Statistical analysis

All statistical analyses were performed using R software (version 4.2.2). Appropriate statistical tests were applied based on data type. Chi-square, Fisher's exact, Mann–Whitney U, Kruskal–Wallis, and Student’s *t*-tests were used where applicable. A two-tailed *P*-value < 0.05 was considered statistically significant.

## Supplementary Information

Supplementary Material 1.

## Data Availability

This study used publicly available data from GEO (https://www.ncbi.nlm.nih.gov/geo). Data on patients from Sun Yat-sen Memorial Hospital of Sun Yat-sen University that were used to support the finding of this study may be released upon application to the corresponding author Herui Yao & Yunfang Yu. The data supporting the findings of this study can be obtained from the corresponding author upon reasonable request. All supplementary materials and relevant data links are thoroughly cited within the manuscript.
