# Supplementary material for: Single-cell RNA sequencing identifies CD8Teff cell activation as a predictive biomarker in triple-negative breast cancer immunotherapy
Source: Mol Biomed. 2025 Sep 19;6:66. doi: 10.1186/s43556-025-00306-2 (PMC12449285; doi:10.1186/s43556-025-00306-2)
Supplement: Supplementary file 1 — Supplementary Material 1. [file 43556_2025_306_MOESM1_ESM.docx]

**Single-Cell RNA Sequencing Identifies CD8Teff Cell Activation as a Predictive Biomarker in Triple-Negative Breast Cancer Immunotherapy**

Luhui Mao^1†^, Zebang Zhang^1†^, Yongjian Chen^2†^, Qing Peng^1†^, Zhenjun Huang^1†^, Wenhao Ouyang^1^, Dongqiang Zeng^3^, Wei Ren^1^, Zifan He^1^, Tang Li ^1^, Zehua Wang^4,5,6^, Ruichong Lin^4,5,6^, Jianli Zhao^1*^, Jiannan Wu^1*^, Herui Yao^1*^, Yunfang Yu^1,4,7,8*^

^1^Guangdong Provincial Key Laboratory of Malignant Tumor Epigenetics and Gene Regulation, Guangdong-Hong Kong Joint Laboratory for RNA Medicine, Department of Medical Oncology, Sun Yat-sen Memorial Hospital, Sun Yat-sen University, Guangzhou, China.

^2^Dermatology and Venereology Division, Department of Medicine Solna, Center for Molecular Medicine, Karolinska Institute, Stockholm, Sweden

^3^Department of Oncology, Nanfang Hospital, Southern Medical University, Guangzhou, Guangdong, P.R. China

^4^Faculty of Innovation Engineering, Institute for AI in Medicine and faculty of Medicine, Macau University of Science and Technology, Taipa, Macao, China.

^5^School of Computer and Information Engineering, Guangzhou Huali College, Guangzhou, China.

^6^UMedEVO and UMedREVO Artificial Intelligence Technology (Guangzhou) Co., Ltd.

^7^Guangdong Provincial Key Laboratory of Cancer Pathogenesis and Precision Diagnosis and Treatment, AI Big Data Laboratory, Shenshan Medical Center, Memorial Hospital of Sun Yat-sen University, Shanwei, China.

^8^Department of Breast Surgery, The First Affiliated Hospital, Jinan University, Guangzhou, China.

^†^These authors contributed equally to this work.

*Co-Corresponding authors: Jianli Zhao, Jiannan Wu, Herui Yao and Yunfang Yu.

**Supplementary Content**

**Figure S1.** CD8A staining demonstrates spatial and expression-specific patterns.

**Figure S2.** Distribution of T cell subsets in triple negative breast cancer.

**Figure S3.** CD8Teff cells demonstrate enhanced tumor killing ability.

**Figure S4.** CD8Teff cells affect antigen presentation and differentiation in immune cells.

**Figure S5.** CD52+DC activates CD8Teff to transform the immune microenvironment.

**Figure S6.** Distribution of cancer cells, fibroblast cells and endothelial cells.

**Figure S7.** CD8Teff cells influence tumor metabolism.

**Figure S8.** CD8Teff cell-based pathology AI for distinguishing tumor type.

**Table S1.** Clinicopathological information of patients.

**Table S2.** Publicly available transcriptomic, single-cell RNA-seq cohorts and clinical cohorts used in this study.

**Table S3.** Experimental reagents.

**Table S4.** Breast tumor cells characteristics.

**Table S5.** Peripheral Blood cells characteristics.

**Figure S1. CD8A staining demonstrates spatial and expression-specific patterns.**


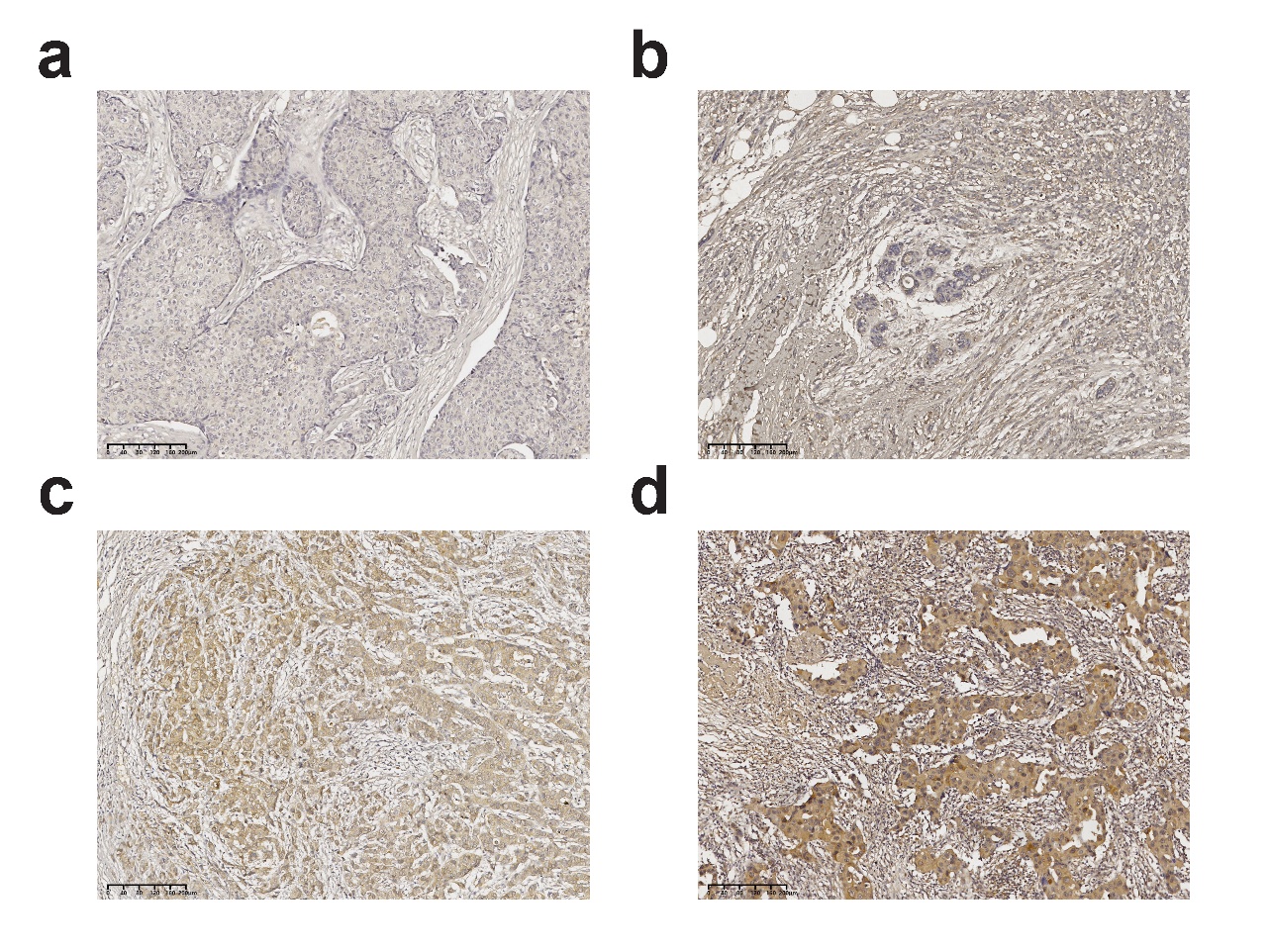


1. Negative, (b) weakly positive, (c)moderately positive and (d) strong CD8A expression.

**Figure S2. Distribution of T cell subsets in triple negative breast cancer.**


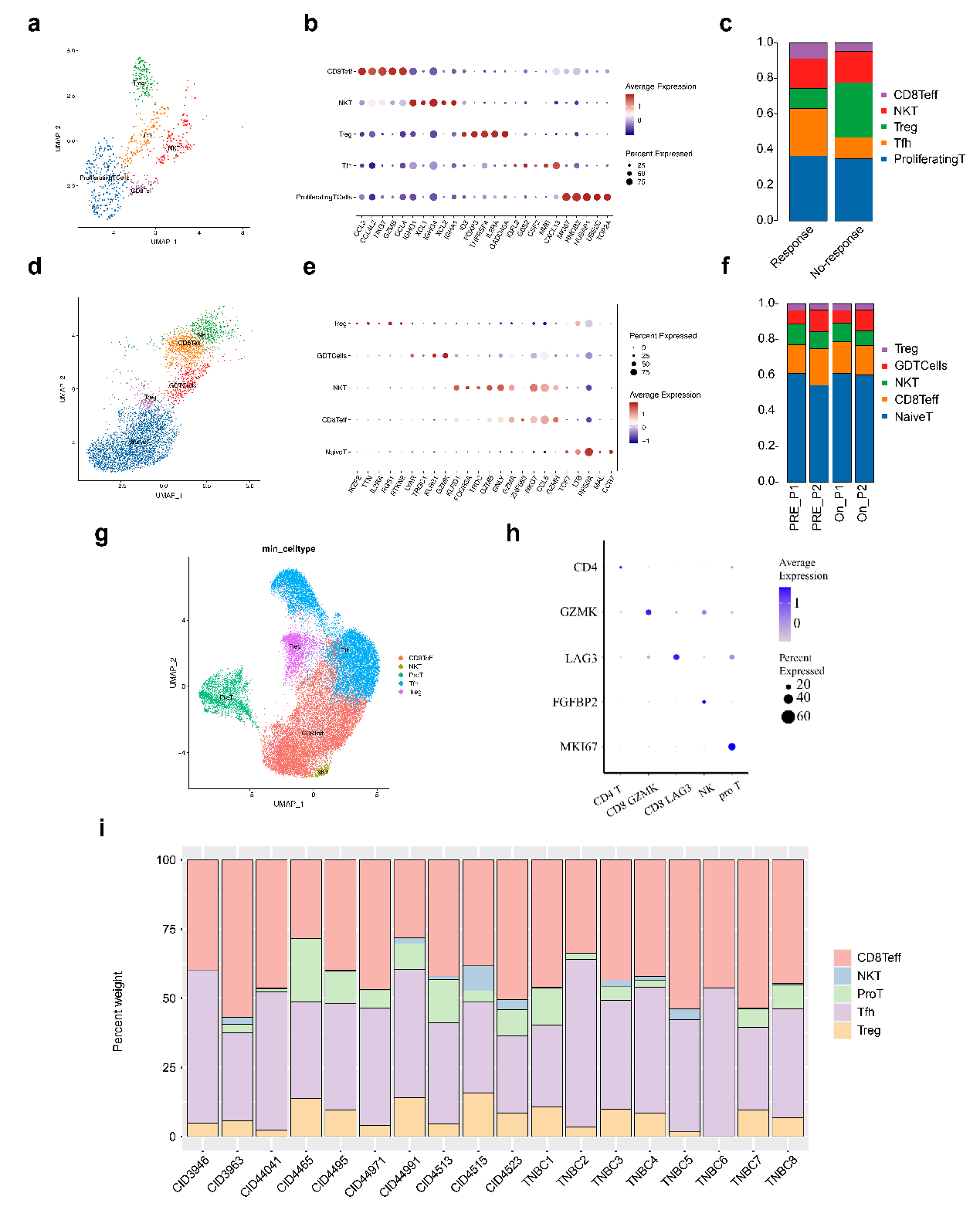


(a) UMAP showing 5 T cell clusters identified in tumor tissue samples using single-cell RNA sequencing (scRNA-seq) data. (b) Dot plot representing marker gene expression across the identified immune cell subsets in tumor tissue. (c) Bar plot depicting the proportions of immune cell populations in tumor tissue samples. (d) UMAP showing 8 T cell clusters identified in peripheral blood samples using scRNA-seq data. (e) Dot plot showing marker gene expression across immune cell subsets in peripheral blood. (f) Bar plot showing the proportions of immune cell populations in peripheral blood samples. (g) UMAP displaying immune cell distribution across 18 TNBC patients from the GSE176078 and GSE161892 cohorts. (h) Dot plot of marker gene expression in immune cells from 18 TNBC patients across the GSE176078 and GSE161892 cohorts. (i) Stacked bar plot showing the distribution of T cell subsets across different clinical groups from the GSE176078 and GSE161892 cohorts.

**Figure S3. CD8Teff cells demonstrate enhanced tumor killing ability.**


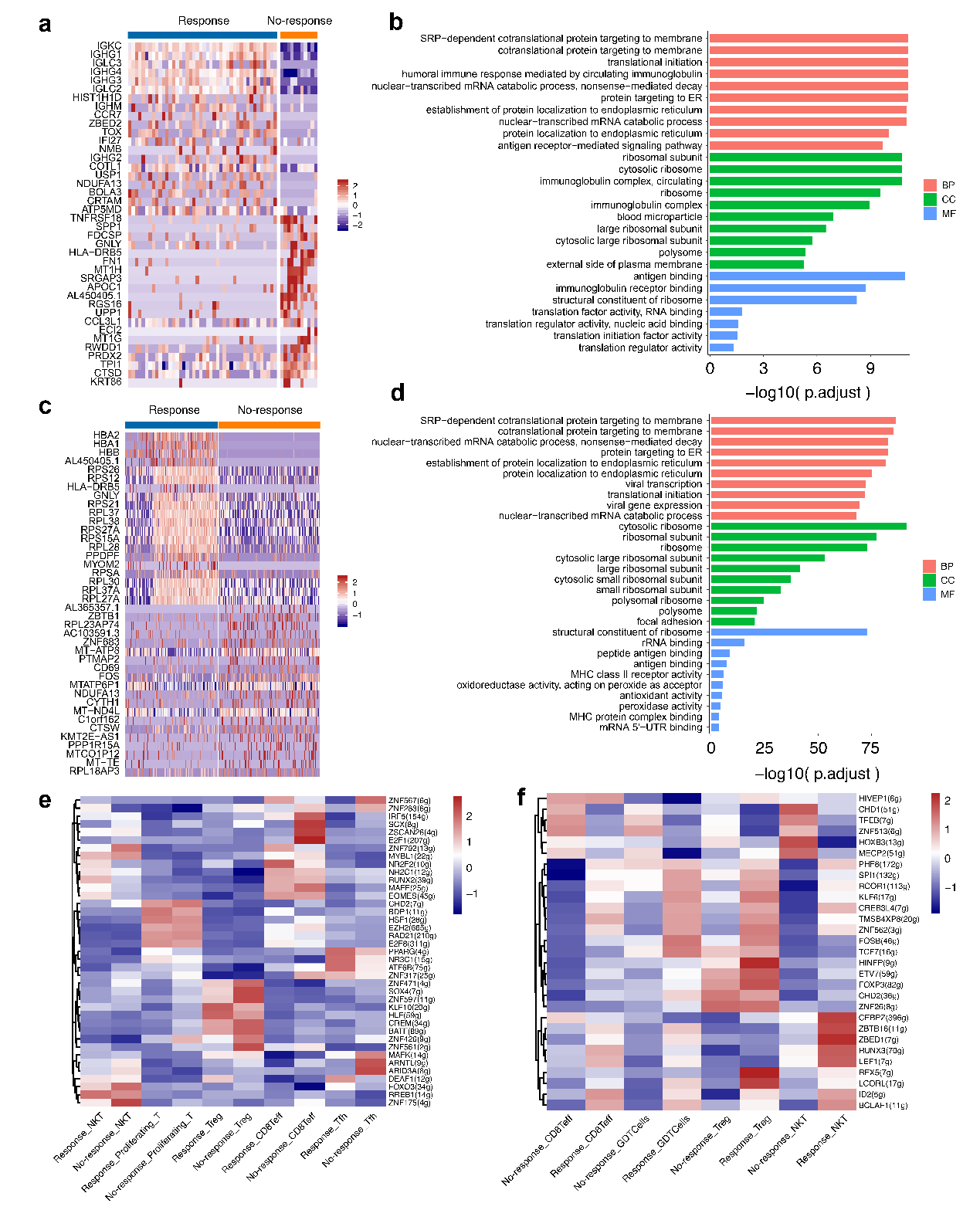


(a) Heatmap showing gene expression differences between response and no-response groups in tumor tissue samples. (b) GO enrichment analysis highlighting key biological processes between response and no-response groups in tumor tissue samples. (c) Heatmap showing gene expression differences between response and no-response groups in peripheral blood samples. (d) GO enrichment analysis highlighting key biological processes between response and no-response groups in peripheral blood samples. Heatmaps showing transcription factor activity analysis across different immune cell types in (e) tumor tissues and (f) peripheral blood samples.

**Figure S4.** **CD8Teff cells affect antigen presentation and differentiation in immune cells.**

**
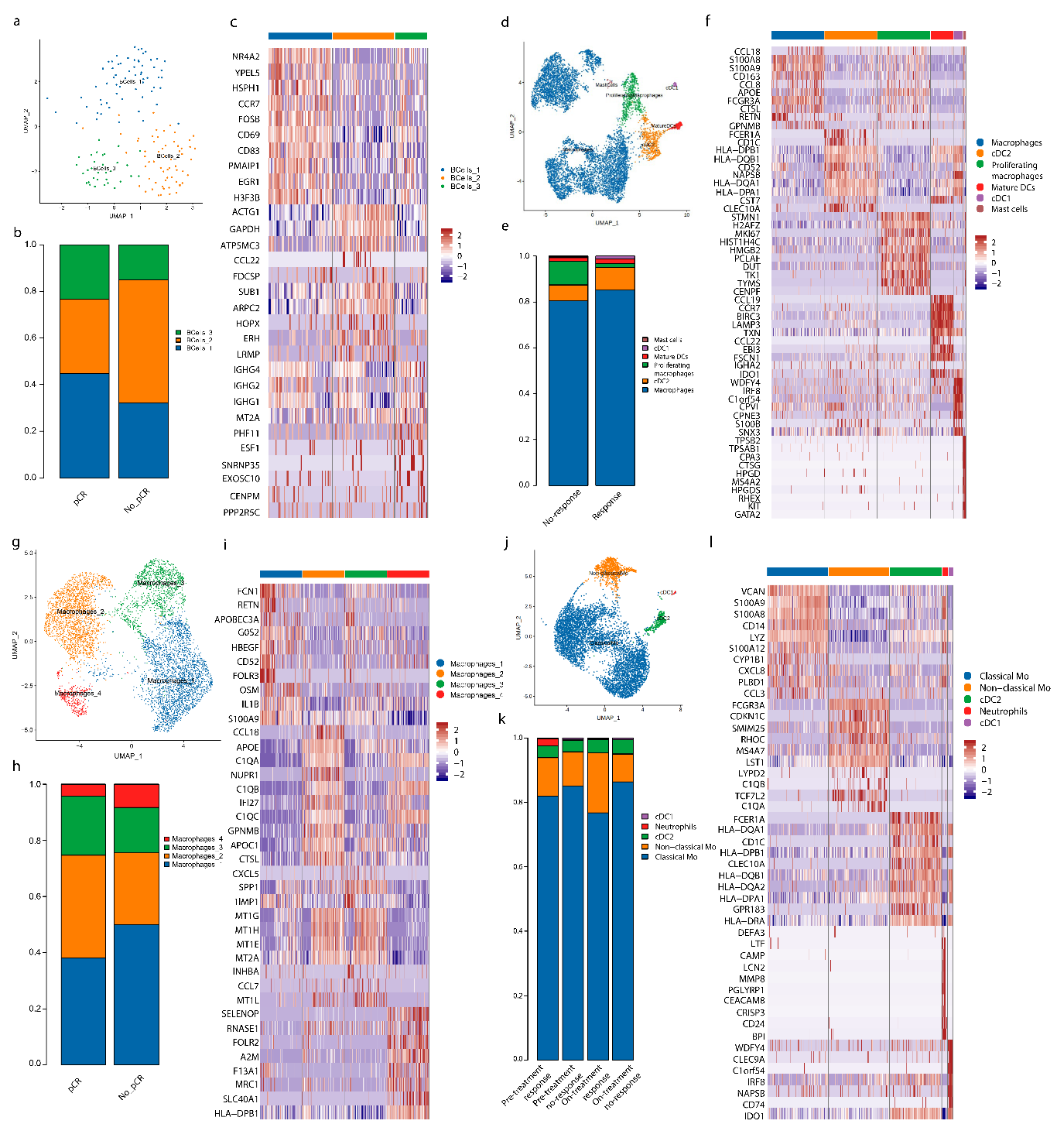
**

(a) UMAP plot showing the distribution of B cells subsets in tumor. (b) Bar plot showing the proportions of B cells populations in tumor. (c) Heatmap displaying gene expression of B cells differences across different subgroups in tumor. (g) UMAP plot showing the distribution of Macrophages subsets in tumor. (h) Heatmap displaying gene expression of Macrophages differences across different subgroups in tumor. (i) Bar plot showing the proportions of Macrophages populations in tumor. UMAP plot showing the distribution of dendritic cell (DC) subsets in (d) tumor and (j) blood samples. Bar plot showing the proportions of these immune cells populations in (e) tumor and (k) blood samples. Heatmap displaying gene expression of these immune cells differences across different subgroups in (f) tumor and (l) blood samples.

**Figure S5. CD52+DC activates CD8Teff to transform the immune microenvironment.**

**
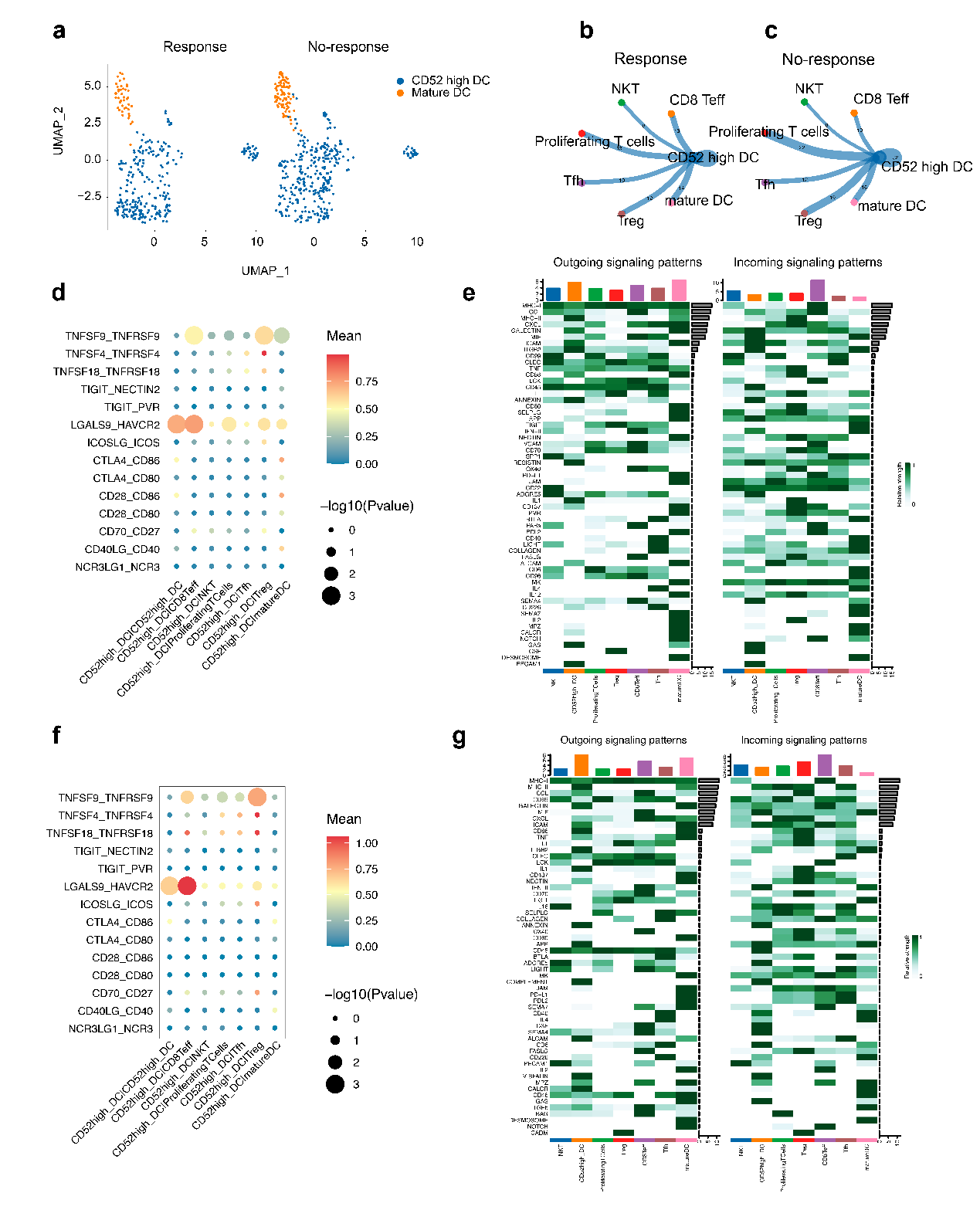
**

(a) UMAP plot showing the distribution of CD52+ DCs and Mature DCs in the tumor microenvironment. Cell-cell communication networks in the (b) response and no-response (c) groups. Dot plots displaying key ligand-receptor interactions involved in immune signaling pathways in the (d) response and no-response (f) groups. Heatmaps showing outgoing and incoming signaling patterns for immune cell populations in the (e) response and no-response (g) groups, emphasizing the role of CD52+ DCs in modulating the immune landscape.

**Figure S6. Distribution of cancer cells, fibroblast cells and endothelial cells.**


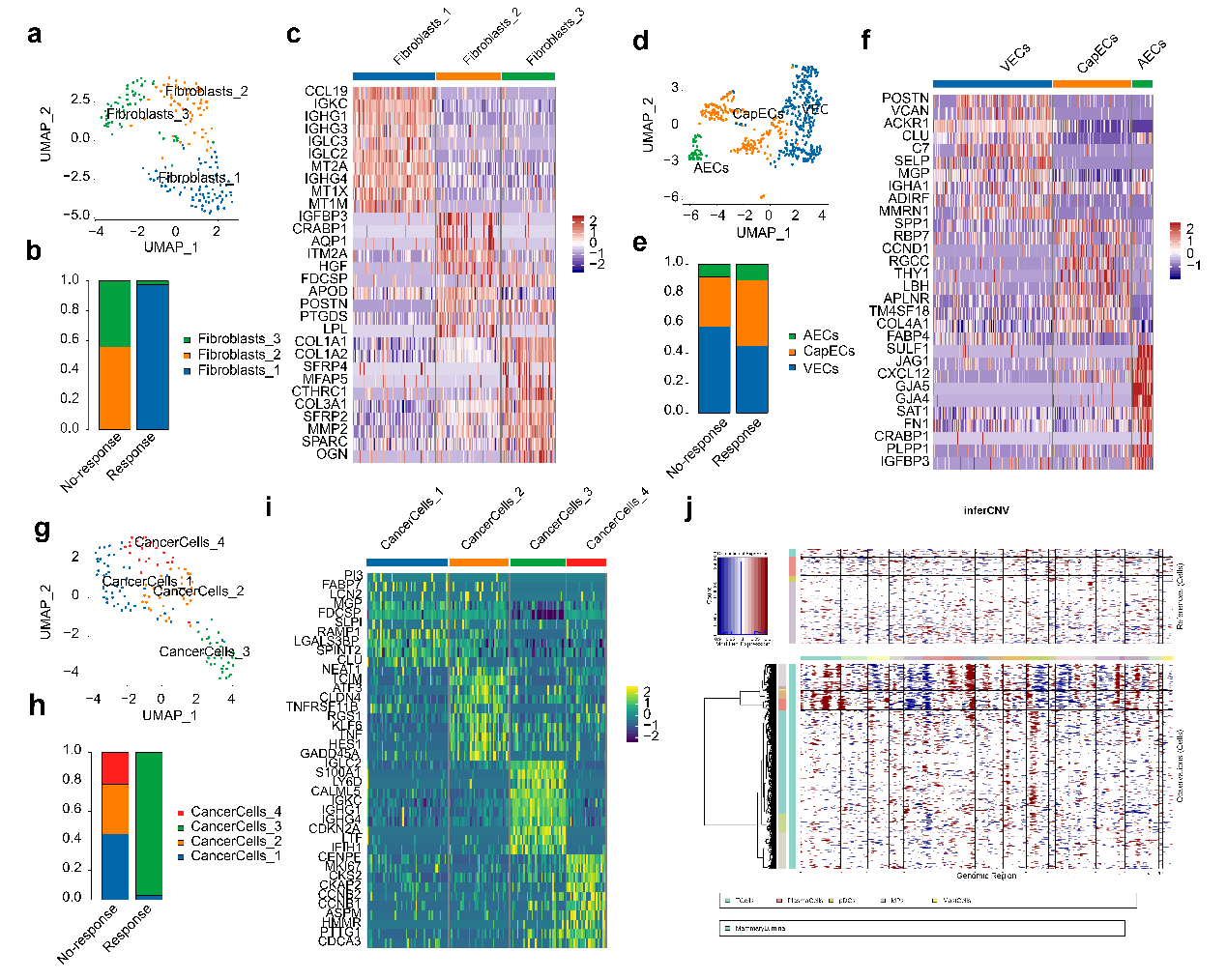


(a) UMAP plot of fibroblast cells showing three subclusters. (b) Bar plot of fibroblast subcluster proportions in the response and no-response groups. (c) Heatmap of gene expression in fibroblast subclusters. (d) UMAP plot of endothelial cell and (e) bar plot of endothelial cell subcluster proportions in the response and no-response groups. (f) Heatmap of gene expression in endothelial cell types. (g) UMAP plot of Cancer cell types. (h) Bar plot of Cancer cell subtype proportions in the response and no-response groups. (i) Heatmap of gene expression in Cancer cell types. (j) Interchromosomal copy number variation (CNV) in cancer cell subclusters.

**Figure S7. CD8Teff cells influence tumor metabolism.**


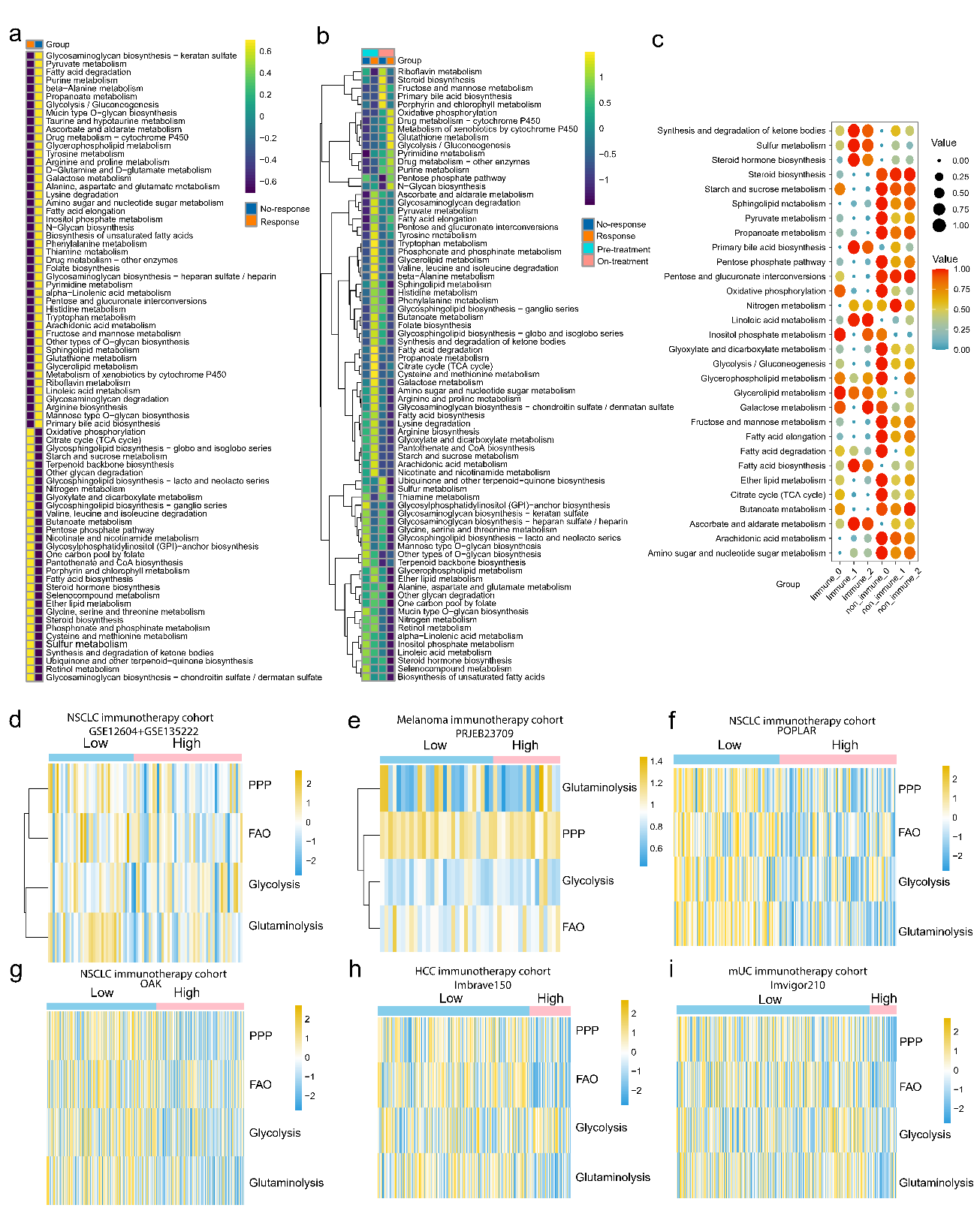


Enrichment analysis of metabolic pathways between response groups and no-response groups respectively in (a) tumor tissues and (b) peripheral blood samples. (c) Enrichment analysis of metabolic pathways among different response groups in 18 TNBC samples. (d–i) Metabolic pathway reprogramming heatmaps (glycolysis, glutaminolysis, FAO, and PPP) between high and low CD8Teff infiltration groups across multiple immunotherapy datasets, including: (d) NSCLC cohort (GSE12604+GSE135222), (e) melanoma cohort (PRJEB23709), (f) NSCLC (POPLAR), (g) NSCLC (OAK), (h) HCC (Imbrave150), and (i) mUC (Imvigor210).

**Figure S8. Characteristics of hot and cold tumor immune microenvironment.**

**
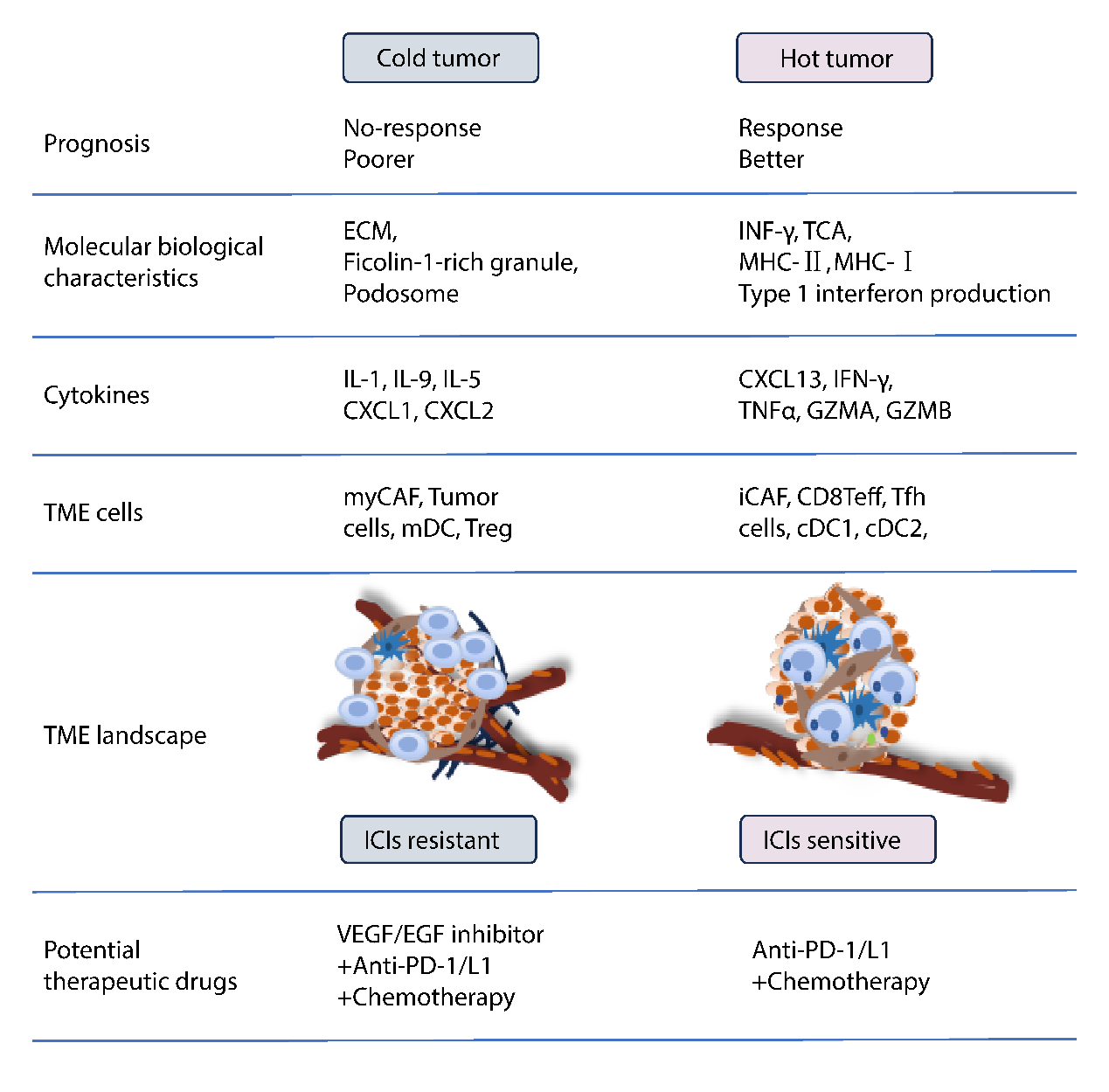
**

ICIs: Immune Checkpoint Inhabitors, ECM: Extracellular Matrix, myCAF: Myofibroblastic Cancer-Associated Fibroblasts, iCAF: Inflammatory Cancer-Associated Fibroblasts, Teff: T Effector Cells, Tfh: T Follicular Helper Cells, mDC: Myeloid Dendritic Cells, Treg: Regulatory T Cells, cDC1/cDC2: Conventional Dendritic Cells Type 1/Type 2.

**Table S1. Clinicopathological information of patients.**

| Patient ID | Treatment | Sex | Tissue | Response |
| --- | --- | --- | --- | --- |
| Patient 1 | Neoadjuvant chemotherapy combined with Camrelizumab | female | Breast cancer tissue | Response (p-CR) |
| Patient 2 | Neoadjuvant chemotherapy combined with Camrelizumab | Female | Breast cancer tissue | No-Response (Non-pCR) |
| Patient 3 | Chemotherapy combined with Camrelizumab | Female | Peripheral Blood (pre-treatment) | Response |
|  |  |  | Peripheral Blood (on-treatment) | Response |
| Patient 4 | Chemotherapy combined with Camrelizumab | Female | Peripheral Blood (pre-treatment) | No-Response |
|  |  |  | Peripheral Blood (on-treatment) | No-Response |

**Table S2. Publicly available transcriptomic, single-cell RNA-seq cohorts and clinical cohorts used in this study.**

| Cohort | Transcriptomic or single-cell RNA-seq URL | Dataset ID | Clinical Data URL | Clinical Dataset ID |
| --- | --- | --- | --- | --- |
| Transcriptomic cohorts | | | | |
| TCGA BRCA | https://portal.gdc.cancer.gov/projects/TCGA-BRCA | N/A | N/A | N/A |
| PRJEB23709 | https://www.ebi.ac.uk/ena/browser/view/PRJEB23709 | N/A | N/A | N/A |
| GSE126044 | https://www.ncbi.nlm.nih.gov/geo/query/acc.cgi?acc=GSE126044 | N/A | N/A | N/A |
| GSE135222 | https://www.ncbi.nlm.nih.gov/geo/query/acc.cgi?acc=GSE135222 | N/A | N/A | N/A |
| Single-cell RNA-seq cohorts | | | | |
| GSE176078 | https://www.ncbi.nlm.nih.gov/geo/query/acc.cgi?acc=GSE176078 | N/A | N/A | N/A |
| GSE161892 | https://www.ncbi.nlm.nih.gov/geo/query/acc.cgi?acc=GSE161892 | N/A | N/A | N/A |
| Clinical cohorts | | | | |
| POPLAR | https://ega-archive.org/datasets/EGAD00001007703 | EGAD00001007703 | https://ega-archive.org/studies/EGAS00001005013 | EGAD00001008548 |
| OAK | https://ega-archive.org/datasets/EGAD00001007703 | EGAD00001007703 | https://ega-archive.org/studies/EGAS00001005013 | EGAD00001008549 |
| Imbrave150 | https://ega-archive.org/datasets/EGAD00001008128 | EGAD00001008128 | https://ega-archive.org/datasets/EGAD00001008130 | EGAD00001008130 |
| Imvigor210 | https://ega-archive.org/studies/EGAS00001004386 | EGAD00001006960 | https://ega-archive.org/studies/EGAS00001004386 | EGAS00001004386 |

**Table S3. Experimental reagents.**

| Multi-label immunofluorescence | | |
| --- | --- | --- |
| LSM800 with airscan laser confocal microscope | Zeiss company | N/A |
| Phosphate buffer Solution (PBS) | Hyclone company | SH30256.FS |
| Slide of glass | Guangzhou Shitai Company | N/A |
| Cover glass slip | Guangzhou Shitai Company | N/A |
| GZMB | CST | 44153 |
| CXCL13 | CST | 85679 |
| CD8A | CST | 85336 |
| CD56 | proteintech | 14255-1-AP |
| TSA | Record Bio | RC0086Plus-34RM-100T |
| Universal two-step test kit (mouse/rabbit enhanced polymer test system) | ZSGC-BIO | PV-9000 |
| Mounting Medium, antifading (with DAPI) | Solarbio | S2110 |

**Table S4. Breast tumor cells characteristics.**

| Cell type | Marker |
| --- | --- |
| Endothelial cells | CDH5, PECAM1, VWF, CLDN5 |
| Mural cells | ACTA2, RGS5, MCAM, MYLK, MYH11 |
| Fibroblasts | DCN, LUM, COL1A1, COL1A2 |
| T cells | CD2, CD3D, TRAC, TRBC1 |
| Mast cells | TPSAB1, TPSB2, CPA3 |
| Mononuclear phagocytes | LYZ, MRC1.CD68, CD14, FCN1, FCGR3A, VCAN, CD1C, CD1E, XCR1, FCER1A, CLEC9A |
| Osteoclasts | ACP5, CTSK, MMP9 |
| Plasmacytoid dendritic cells | IL3RA, CLEC4C, LILRB4, GZMB |
| Plasma cells | CD79A, JCHAIN, MZB1, IGHG1 |
| Basal cells | KRT17, KRT14, KRT5, TP63 |
| Luminal epithelial cells of mammary | KRT18, KRT8, KRT19, FOXA1, MUC1 |

**Table S5. Peripheral blood cells characteristics.**

| Cell type | Marker |
| --- | --- |
| T cells | CD3D, CD2, TRAC, TRBC2, KLRD1 |
| Neutrophils | CSF3R, CXCR2, FCGR3B |
| B cells | MS4A1, CD79A, CD79B |
| Plasma cells | CD79A, JCHAIN, MZB1 |
| Platelets | PPBP, PF4, TUBB1, GP9 |
| Erythrocytes | HBB, HBA1, ALAS2, SNCA, CA1 |
| Mononuclear phagocytes | LYZ, CD14, VCAN, CD1C, CD1E, FCER1A, XCR1 |
| Basophils | CLC, GATA2, CPA3, MS4A2 |
